# Supplementary material for: Social Determinants of Health: A Multilingual Standardized Patient Case to Practice Interpreter Use in a Telehealth Visit
Source: MedEdPORTAL. 2023 Nov 14;19:11364. doi: 10.15766/mep_2374-8265.11364 (PMC10643468; doi:10.15766/mep_2374-8265.11364)
Supplement: Supplementary file 1 — SP Case - Spanish.docxSP Case - Tagalog.docxSP Case - Igbo.docxSP Case - French.docxSMI - Spanish.docxSMI - Tagalog.docxSMI - Igbo.docxSMI - French.docxSPL Rehearsal Script.docxDoor Instructions - Spanish and Tagalog.docxDoor Instructions - Igbo.docxDoor Instructions - French.docxFaculty Guide.pdfStudent Guide.pdfImportant Points Interpreters Telehealth.docxGraphic Instructional Tool.pdfSample Progress Note.docxProgress Note Grading Rubric.xlsx [file mep_2374-8265.11364-s001.zip › B. SP Case - Tagalog.docx]

**Appendix B: Standardized Patient Case Development Tool**

Date: April 1, 2020 (revised June 14, 2023)

Primary Case Author: Gigi Guizado de Nathan

Case Translator: Marie Christine Padilla (Tagalog translation)

Standardized Patient Educator: Brenda Lopez, Brandi Blackman, Timothy Cummings

Name of Case: Beto or Berta Ruiz – Tagalog Version

Name of educational and or assessment activity: UNLVSOM Doctoring 2 week 13

Patient Name: Beto or Berta Ruiz

Chief Complaint: Extreme fatigue

Most likely Diagnosis and Differential with rationale from history and/or physical exam: COVID-19, influenza, SARS-CoV-2

Challenge question:

Domains: Check all that apply

Professionalism

X Communication and Interpersonal skills

X Medical History

Physical exam

Shared Decision Making

Patient Education

X Clinical Reasoning

Documentation

Handoff

Presentation

X Other: Interpreter

Type and level of learner:

Case Objectives: please list specific objectives for each of the domains you have checked above:

1. Develop ways to create an environment conducive to conducting a telehealth visit that includes an interpreter.
2. Demonstrate appropriate history gathering and physical exam components while interviewing a patient with fatigue during a telehealth visit.
3. Apply techniques from the interpreter services reference materials to interview a non-English language preference patient with an interpreter and critique a peer after observing.
4. Integrate information from the case and faculty and peer feedback to create a progress note with an appropriate basic differential diagnosis and treatment plan for a patient with fatigue.

SPECIAL NEEDS/EQUIPMENT (over and above standard exam room set-up):

1. Computer devices with internet access (desktop computer with monitor, laptop computer, tablet, smartphone, etc.) for learner(s) and two standardized patients.

2. ZOOM, Google Hangouts, Webex, Skype, Facetime, or other online platform for telehealth meetings.

| SETTING: outpatient, in patient, ED, home, nursing home, rehab, group etc. | Beto/Berta Ruiz is an adult male or female who has been told to call TELEHEALTH SERVICES today for extreme fatigue. The patient does not speak English.  The patient is a non-English speaking man or woman who is complaining of extreme fatigue. The patient called the doctor’s office, was told to stay home and was given a telehealth appointment. The patient requires a translator during the appointment. |
| --- | --- |
| PATIENT PROFILE: Information about the “patient” that helps select an SP and helps the learner get an understanding of them as a person. SP will know more information about the patient than learner will ever ask but allows SP to portray a fully developed patient personality. If none of the items below are particulars for the case please write “all may be used.” | |
| Age range | Adult |
| Race and/or ethnic group | Asian, Pacific Islander, Southeast-Asian, or mixed race |
| Religious/spiritual background | Ako at ang aking mga anak ay pinalaking Catolico. (I was raised Catholic and raised my kids Catholic.) |
| Sex (e.g., male, female, intersex, transwoman, transman) | Any |
| Sexual Orientation (e.g., heterosexual, lesbian, gay, bisexual, pansexual, queer, asexual) | Any |
| Gender expression (e.g., man, woman, gender queer) | Any |
| Race/ethnicity: | Asian, Pacific Islander, Southeast Asian, or mixed race |
| Physical description (e.g., BMI, height range) | Any |
| Physical limitations (e.g.,) | none |
| Patient appearance (e.g., disheveled, hospital gown, business casual, casual) | Street clothes |
| Moulage + location (e.g., none, bruises, scars, body piercing, tattoos) | None |
| Affect (e.g., pleasant, cooperative) | In general, Beto/Berta, and the translator, are pleasant and easy to talk to. You answer all questions directly without ‘dancing around the subject’. Beto/Berta will cough (into their elbow or a tissue) at the start of the encounter. S/He will also appear fatigued throughout the encounter. |
| Family group (e.g., who is family, who they live with) | Ako ay nakatira sa isang bahay sa Las Vegas. Kasama ko ang aking asawa at dalawang anak na nasa tamang edad na. (You live in a house in Las Vegas with your spouse. Your 2 children are grown.)  Malaki ang aking support system. Mula sa aking mga malapit na pamilya at kamag-anak hanggang sa aking mga kaibigan na tinuturing ko narin na pamilya. (Large support system of close-knit family and friends who are like family.) |
| Education | Enough to establish & successfully run a business. |
| Level of health literacy | Low |
| Employment, if any - present and past, noting any current stresses | Ako ay isang restaurant owner. Sana di ako mawalan nang panghanapbuhay. (I own a restaurant. I hope we don’t go out of business.) |
| Home/homeless - type of dwelling, number of stories, owned or rented | Ako ay nakatira sa isang bahay sa Las Vegas. Kasama ko ang aking asawa at dalawang anak na nasa tamang edad na. (You live in a house in Las Vegas with your spouse. Your 2 children are grown.) |
| Financial situation- any current stresses | Ako ay isang restaurant owner. Sana di ako mawalan nang panghanapbuhay. (I own a restaurant. I hope we don’t go out of business.) |
| Insurance Status (e.g., un/under/insured, public/private, HMO/PPO) | insured |
| Habits (i.e., diet, exercise, caffeine, smoking, alcohol, drugs) | Alcohol : tatlo hanggang apat na inom bawat lingo (3-4 drinks a week)  Tobacco : Hindi, kahit kailan. (No, never)  Diet : Ako ay kumakain nang masustansya at balanceng diyeta. Lahat sa aking pamilya ay magaling magluto. (I eat a balanced diet at the restaurant and at home. There are nothing but great cooks in my family.)  Caffeine : Isang tasa bawat umaga, pang agahan. (1 cup each morning with breakfast)  Exercise : Iniingatan ko ang aking kalusugan, lagi akong nag-la-lakad, at palagi akong gumagalaw habang nasa trabaho. (I take care of myself, I take walks, and I’m on my feet all day at work.) |
| Activities (i.e., hobbies, sports, clubs, friends) | Mahilig akong mag-spend time sa aking pamilya. Nakikipaglaro sa aking mga apo. Nag-a-attend nang soccer games nang aking pamilya. Spending time with my family. Playing with my grandkids. Going to my family’s soccer games.) |
| Typical day - what is the usual daily routine |  |

| CASE INFORMATION | |
| --- | --- |
| Chief Concern: What the patient will say when greeted by the student. The patient’s primary reason for seeking medical care often stated in his/own words. | “Ako ay pagod at nanghihina. Nahirapan nga akong maglakad pabalik galing sa tindahan. <umubo>” (I am so tired and weak. I barely made it back from my usual walk to the corner store. <cough>) |
| Additional Concerns: Other, if any, concerns the patient has today (i.e., symptoms, requests, expectations, etc.) that will become part of set agenda. | “Nagduda nga ako kung makakalakad pa akong pabalik nang bahay para makabalik sa aking kama. <pag tinanong, mga 15 lakad kalayo> (I really doubted I was gonna make it from my front door to my bed. < if asked, it’s 15 paces or so >)  “Gusto ko na talagang makabalik sa trabaho.” (I’d really like to get back to work.) |
|  | |
| THE PATIENT STORY: The SP will be asked to tell their symptom story and the personal and emotion impact for each of their concerns. You will want to write this is the patient voice. The symptom story should be able to answer this question:“Tell me more about [chief concern/additional concern], starting at the beginning and bringing me up to now.”  The personal context should be able to answer questions concerning the broader personal/psychosocial context of symptoms, especially the patient beliefs/attributions.  The emotional context should be able to ask how are you doing with this, how does this make you feel, how has this affected you emotionally? IMPACT: How has this affected your life? How has this been for your family? | You are a non-English speaking person (use your own age and gender) who is complaining of extreme fatigue. Your bilingual spouse called the doctor’s office and was given a telehealth appointment. You need a translator for this appointment, as your spouse has gone to work, holding down the family restaurant.  You and your extended family own and operate Don Tortaco restaurants in Las Vegas and North Las Vegas. Today is the third day in a row that you have stayed home with fatigue, fever, chills and cough. In the past 24 hours, diarrhea and runny nose have started, too. As a result, you’ve lost your appetite. You’ve had the flu before, but the severity of the fatigue is new to you. You have never felt so sick in your life.  Two weeks ago, you and your spouse returned from a dream vacation to Spain. Three days ago, the extreme fatigue set in while you were on a typical walk to the corner store.  Fever, chills, and a dry cough soon followed.  You haven’t had much appetite since the runny nose started yesterday, along with the unsettled stomach and diarrhea.  Your spouse is concerned about your health. Deep down, you are too.  Perhaps because it is easier on you emotionally and psychologically, you are remaining focused on keeping your business alive. |
| HISTORY OF PRESENT ILLNESS: Although some of the HPI will be given in the patient’s symptom story, the learners will expand the story during the direct question section. Below describe the detailed history, usually about the chief concern, which the student must develop in order to make a useful assessment of the problem: | |
|  | |
| Onset (when; gradual or sudden) | Ako ay nanghihina, mga tatlong araw na. (The fatigue started about 3 days ago.) |
| Setting (what was going on or where was patient when symptoms first noticed?) |  |
| Duration (how long) | Tatlong araw. (3 days) |
| Time relationships (frequency, constant or intermittent) | Palage. (Constant) |
| Location | Nananakit ang buong katawan ko. (My whole body aches.) |
| Radiation | Wala. (None) |
| Quality | Ako ay sobrang nanghihina, ni hindi ko kayang magbasa nang dyaryo habang nakahiga.  (I’m too weak to even read the newspaper while I’m in bed.) |
| Amount | Sa buong buhay ko, ngayon lang ako nagkasakit nang ganito. (I’ve never felt so sick in my life.) |
| Aggravated by what | Mas lalo akong nanghihina pag tumatayo ako, papuntang banyo. (Getting up to go to the bathroom makes it worse.) |
| Relieved by what | Walang nakakatulong sa panghihina kong ito. (Nothing makes the fatigue better.) |
| Associated with what | Lagnat, panlalamig, pananakit nang katawan, ubo, pagtatae, sipon. (Fever, chills, body aches, cough, diarrhea, runny nose.)  If asked, the constant fever ranges from 100 to 102, the cough is constant, the diarrhea is watery and happens about 4 times a day. |
| Attitude (what does the patient think is the problem, and how does he/she feel about it) | Gustong gusto ko nang bumalik sa trabaho. (I want to get back to work as soon as possible.)  Hindi ako makapagtrabaho. Lahat nang kabuhayan ko, nang pamilya ko, ng mga empleyado ko ay nakasalalay sa akin.  (I can’t go to work. The future of my business, and the livelihoods of my family and employees are all depending on me.) |
| Overall course |  |
| REVIEW OF SYSTEMS: Significant positives and negatives | |
| GENERAL: Body aches. | GI: Diarrhea and loss of appetite. |
| ENT: Runny nose. | ENDOCRINE: Chills and fever. |
| LUNG: Dry cough. | NEUROLOGIC: Weakness. |
|  |  |
|  | |
| Past medical history | Wala. Noon paman, di ako nagkakasakit. (None, I’ve always been healthy.)  Wala naman masyado, siguro mga sugat bata lang noon, etc. (Nothing major, occasional childhood sprains, etc.) |
| Medication allergies (Name and reaction) | Wala (None) |
| Environmental allergies (Name and reaction) | Wala (None) |
| Illnesses | Wala. Noon paman, di ako nagkakasakit. (None, I’ve always been healthy.) |
| Vaccinations | Hindi ako nakakakuha ng taunang bakuna laban sa trangkaso at hindi ako nakakuha ng anumang pagbabakuna sa COVID.  (I do not get an annual flu shot and did not get any COVID vaccinations.) |
| Surgeries | Wala (None) |
| Accidents/ injuries/ trauma | Wala naman masyado, siguro mga sugat bata lang noon, etc. (Nothing major, occasional childhood sprains, etc.) |
| Hospitalization | Wala (None) |
|  | |
| Inclusive sexual and reproductive history | |
| Sexual practices  Sexual partners  Protection: Use of safer sex practices  Use of birth control if appropriate  Risk of intimate partner violence | Ako at nang asawa ko ay actibo sa pagtatalik.  (I’m active with my spouse.) |
| Ob/GYN HISTORY | Age of onset of menses N/A  Age of menopause N/A  Number of pregnancies N/A  Number of live births N/A  Number of miscarriages N/A  Number of abortions N/A |
| Medications | Prescription/dose/reason: Wala (None)  Over the counter/dose/reason: Tylenol lang para sa paglalagnat at pananakit nang katawan. <Kapag tinanong, Sa aking palagay, ito ay nakakatulong nang konti, kahit papano.> (Tylenol for the fever and body aches. If asked whether it helps, I think it helps a little.) You’ve been taking Tylenol according to the directions on the box (2 pills every 6-8 hours) since the fever began. The fever peaks at 102 and the Tylenol brings it down to 100.  Herbs/supplements/dose/reason: Wala (None)  Other: N/A |
| Immunizations | Hindi ako nakakakuha ng taunang bakuna laban sa trangkaso at hindi ako nakakuha ng anumang pagbabakuna sa COVID.  (I do not get an annual flu shot and did not get any COVID vaccinations.) |
| Tobacco products:   - Cigarettes - Cigar - Pipe - Chew - E-cigarettes | Hindi, kahit kailan. (No, never) |
| Alcohol   - Beer - Wine - Liquor - Other | Current – 3-4 tragos a la semana  (3-4 drinks a week) |
| Drugs   - Weed - Cocaine - Heroin - Meth - Other - IV - Inhalants - Other | Hindi, kahit kailan. (No, never) |
| Diet (describe) | Ako ay kumakain nang masustansya at balanceng diyeta. Lahat sa aking pamilya ay magaling magluto. (I eat a balanced diet at the restaurant and at home. There are nothing but great cooks in my family.) |
| Exercise (describe) | Iniingatan ko ang aking kalusugan, lagi akong nag-la-lakad, at palagi akong gumagalaw habang nasa trabaho. (I take care of myself, I take walks, and I’m on my feet all day at work.) |
| List any other important social history or information important to this case | Sleep: Nakatulog ako nang mahimbing. Palagi naman akong natutulog nang mga anim hanggang walong tulog bawat gabi. (I haven’t had any trouble sleeping lately. I’ve always slept well, 6 -8 hours each night)  Travel : Ako at nang asawa ko ay kababalik palamang nang Spain mga dalawang lingo na. (My husband/wife and I returned from Spain 2 weeks ago.)  If asked about sick contacts, i.e. Have you been around anyone who is sick? At home? At work?, you reply: Sa pagkakaalam ko, di ako nakipaghalubilo sa taong may sakit, subalit ito ay posible. Dahil, kapag may nakikita akong taong may sipon, humahaching, umuubo, inakala kong may allergy lang sila. Ngayon, hindi na ako sigurado… (Not that I’m aware of, but it’s possible. When I see someone with a runny nose, sneezing, coughing, I usually just figure it’s allergy season. Now I’m not so sure…) |
| Family history |  |
| Mother, Father, Siblings, Grandparents, and other significant findings. | Sa aking pagkakaalam, wala sa aking pamilya ang may malubhang sakit. Kami ay malusog. Lahat kami ay masipag at nag-e-enjoy lang sa buhay hanggang sa mamatay na sa pagtanda. (You are not aware of any major health issues in your family. “I come from a very healthy family. We work hard and play hard until we die of old age.)  As the ages of the SPs portraying this case will vary, so will the ages and health status of their relatives. Please take time to fill in this portion with the ages and health status (either “Alive and Healthy” or “Deceased of Old Age”) of your imaginary family in keeping with your real age. |
|  |  |
| Physical Exam- List exam maneuvers expected for this case and any abnormal findings that SP will simulate. (tenderness, hyper-hypo reflex, rebound, weakness etc. )  Beto/Berta will cough (into their elbow or a tissue) at the start of the encounter. S/He will also appear fatigued throughout the encounter.  There is no physical examination during this case. | |
| PHYSICAL EXAM FINDINGS |  |
| 1. Written in layman’s terms |  |
| 1. General appearance- affect, appearance, position of patient at opening (i.e. sitting, laying down, holding abdomen etc.) | When the student joins the video call you should be sitting in a chair wearing your regular clothes. |
| 1. Vital signs | T: 102° F oral  Pulse: 75 bpm  BP: 132/64  RR: 25 |
| 1. Specific findings and affect | Beto/Berta will appear fatigued throughout the encounter. |
| 1. Response to certain physical movements | Beto/Berta will cough (into their elbow or a tissue) at the start of the encounter. |
|  |  |
| DIAGNOSIS AND DIFFERENTIAL | Diagnosis 1: COVID 19 related illness  History supporting: Dry cough, fever, recent travel |
| Diagnosis with support from positive and negative history and PE findings | 2nd or 3rd Dx : Influenza, bacterial pneumonia, mycoplasma  History supporting: No flu shot this year, dry cough  PE supporting: fever, mildly ill appearing, frequent cough during interview |
| Differential with support from positive and negative history and PE findings |  |
|  | labs/imaging: COVID 19 test, influenza test,cbc, tsh  labs/imaging: chest x-ray if worsens |
| MANAGEMENT OR DIAGNOSTIC PLAN | medications/treatments: Tylenol, motrin, cough meds  education: masks, covering face, cdc.gov  disposition: when to return to work, quarantine protocol, ED precautions |
|  | Cultural competence and working with an interpreter |
| PROFESSIONALISM ISSUES OR CHALLENGES: | Diagnosis 1: COVID 19 related illness  History supporting: Dry cough, fever, recent travel |
