# Supplementary material for: Social Determinants of Health: A Multilingual Standardized Patient Case to Practice Interpreter Use in a Telehealth Visit
Source: MedEdPORTAL. 2023 Nov 14;19:11364. doi: 10.15766/mep_2374-8265.11364 (PMC10643468; doi:10.15766/mep_2374-8265.11364)
Supplement: Supplementary file 1 — SP Case - Spanish.docxSP Case - Tagalog.docxSP Case - Igbo.docxSP Case - French.docxSMI - Spanish.docxSMI - Tagalog.docxSMI - Igbo.docxSMI - French.docxSPL Rehearsal Script.docxDoor Instructions - Spanish and Tagalog.docxDoor Instructions - Igbo.docxDoor Instructions - French.docxFaculty Guide.pdfStudent Guide.pdfImportant Points Interpreters Telehealth.docxGraphic Instructional Tool.pdfSample Progress Note.docxProgress Note Grading Rubric.xlsx [file mep_2374-8265.11364-s001.zip › E. SMI - Spanish.docx]

**Beto/Berta Ruiz – Spanish Version**

***SP Educator information***

Presenting complaint: Extreme fatigue

Differential diagnoses: COVID-19, influenza, SARS-CoV-2

Patient demographics:

Age: Any

Sex: Any

Race: Any/Hispanic

Height: Any

Weight: Any

Physical Findings the SP should NOT have (scars, etc. ): None

PROFILE

The patient is a non-English speaking man or woman who is complaining of extreme fatigue. The patient called the doctor’s office, was told to stay home and was given a telehealth appointment. The patient requires a translator during the appointment.

OBJECTIVES: history/physical/other:

- Develop ways to create an environment conducive to conducting a telehealth visit that includes an interpreter.
- Demonstrate appropriate history gathering and physical exam components while interviewing a patient with fatigue during a telehealth visit.
- Apply techniques from the interpreter services reference materials to interview a non-English language preference patient with an interpreter and critique a peer after observing.
- Integrate information from the case and faculty and peer feedback to create a progress note with an appropriate basic differential diagnosis and treatment plan for a patient with fatigue.

SPECIAL NEEDS/EQUIPMENT (over and above standard exam room set-up):

1. Computer devices with internet access (desktop computer with monitor, laptop computer, tablet, smartphone, etc.) for learner(s) and two standardized patients.
2. ZOOM, Google Hangouts, Webex, Skype, Facetime, or other online platform for telehealth meetings.

PURPOSE OF THIS ACTIVITY:

To practice interviewing a non-English language preference patient in a telehealth setting with an interpreter.

LAST USE OF THIS CASE:

April 22, 2021 for UNLVSOM Doctoring 2 week 13 (revised 6/14/2023)

**CASE AUTHORS:**

Gigi Guizado de Nathan, BA

Translation from the English by Brenda Lopez and Gigi Guizado de Nathan

PRESENTING SITUATION

and

INSTRUCTIONS TO THE STUDENT

Beto/Berta Ruiz

Beto/Berta Ruiz is an adult male or female who has been told to call TELEHEALTH SERVICES today for extreme fatigue. The patient does not speak English.

Vital signs:

T: 102° F oral Pulse: 75 bpm BP: 132/64 RR: 25

You are to:

- Develop ways to create an environment conducive to conducting a telehealth visit that includes an interpreter.
- Demonstrate appropriate history gathering and physical exam components while interviewing a patient with fatigue during a telehealth visit.
- Apply techniques from the interpreter services reference materials to interview a non-English language preference patient with an interpreter and critique a peer after observing.
- Integrate information from the case and faculty and peer feedback to create a progress note with an appropriate basic differential diagnosis and treatment plan for a patient with fatigue.

***SP Information***

Beto/Berta Ruiz

TRAINING MATERIALS

CASE SUMMARY

You are a non-English speaking person (use your own age and gender) who is complaining of extreme fatigue. Your bilingual spouse called the doctor’s office and was given a telehealth appointment. You need a translator for this appointment, as your spouse has gone to work, holding down the family restaurant.

You and your extended family own and operate Don Tortaco restaurants in Las Vegas and North Las Vegas. Today is the third day in a row that you have stayed home with fatigue, fever, chills and cough. In the past 24 hours, diarrhea and runny nose have started, too. As a result, you’ve lost your appetite. You’ve had the flu before, but the severity of the fatigue is new to you. You have never felt so sick in your life.

Two weeks ago, you and your spouse returned from a dream vacation to Spain. Three days ago, the extreme fatigue set in while you were on a typical walk to the corner store. Fever, chills, and a dry cough soon followed.

You haven’t had much appetite since the runny nose started yesterday, along with the unsettled stomach and diarrhea.

Your spouse is concerned about your health. Deep down, you are too. Perhaps because it is easier on you emotionally and psychologically, you are remaining focused on keeping your business alive.

Your challenge, as the standardized patient, is:

1. To appropriately and accurately reveal the facts of the patient’s case through an interpreter in a telehealth setting.

PRESENTATION/EMOTIONAL TONE

When the student joins the video call you should be sitting in a chair wearing your regular clothes.

In general, Beto/Berta, and the translator, are pleasant and easy to talk to. You answer all questions directly without ‘dancing around the subject’. Beto/Berta will cough (into their elbow or a tissue) at the start of the encounter. S/He will also appear fatigued throughout the encounter.

OPENING LINE

In response to the typical opening question from the student, “What brings you in here today?” – you respond verbatim.

**“Estoy tan cansado/a y débil. Apenas volví de mi caminata habitual a la tienda de la esquina. <tos>”**

(I am so tired and weak. I barely made it back from my usual walk to the corner store. <cough>)

If invited to say more:

**“Realmente dudaba si iba a llegar desde la puerta de mi casa hasta mi cama.** <Si me lo piden, **son 15 pasos más o menos>** (I really doubted I was gonna make it from my front door to my bed. < if asked, it’s 15 paces or so >)

If invited to say more:

**“Realmente me gustaría volver al trabajo.”** (I’d really like to get back to work.)

**HISTORY OF PRESENT ILLNESS (HPI)**:

*Onset:* **La fatiga empezó hace unos 3 días.**

*Duration:* **3 días.** 3 days

*Frequency:* **Constante.** Constant

*Quality/Description:* **Estoy demasiado débil para siquiera leer el periodico mientras estoy en la cama.** (I’m too weak to even read the newspaper while I’m in bed.)

*Severity/intensity:* **Nunca en mi vida me he sentido tan enfermo/a.** (I’ve never felt so sick in my life.)

*Location:* **Me duele todo el cuerpo.** (My whole body aches.)

*Aggravating /alleviating factors:* **Lo empeora levantarme para ir al baño. Nada mejora la fatiga.** (Getting up to go to the bathroom makes it worse. Nothing makes the fatigue better.)

*Associated symptoms:* **Fiebre, escalofríos, dolores corporales, tos, diarrhea, nariz que moquea.** (Fever, chills, body aches, cough, diarrhea, runny nose.) If asked, the constant fever ranges from 100 to 102, the cough is constant, the diarrhea is watery and happens about 4 times a day.

**RESPONSE DURING PHYSICAL EXAMINATION:** (ROM, pain, procedure responses during PE to make case clinically accurate): N/A, There is no physical examination during this case.

**Your chief concern / patient perspective of illness** (If the student asks, “What concerns you most about this?” (Or something of that nature), you reply that:

**Quiero volver a trabajar lo antes posible.**

(I want to get back to work as soon as possible.)

If the student asks, “what effect does this have on your daily life?**” (**Or something of that nature, you reply that:

**No puedo ir a trabajar. El futuro de mi negocio y los medios de vida de mi familia y mis empleados dependen de mí.** (I can’t go to work. The future of my business, and the livelihoods of my family and employees are all depending on me.)

**REVIEW OF SYSTEMS** (Items in **bold** indicate a “yes” response)

**GENERAL** – No tearing or redness of the eyes noted. **Body aches.**

**Head** – No headache

**ENT** – No tinnitus (ringing in ears), no loss of hearing. No sensitivity to noises. **Runny nose.**

**EYES:** No loss of vision, no light sensitivity**.** Past examination (in the last year) was normal.

**CV**- no chest pain, no palpitations

**LUNG** –No hemoptysis or wheezing. **Dry cough**.

**Genitourinary** – No problems with urination. No blood in the urine. Male: No erectile dysfunction. Female:(see menstrual history below**).**

**GI** – no abdominal pain. No problems with bowels, no constipation, no nausea. **Diarrhea and loss of appetite.**

**MUSCULOSKELETAL** – No joint pain or muscle pain /spasm.

**ENDOCRINE**- No hot flashes, hair loss or temperature sensitivity, no increased thirst, no recent weight loss. **Chills and** **fever**.

**SKIN**: No new rashes or other problems

**NEUROLOGIC**: No numbness, tingling, tremor, fainting, memory loss or loss of balance. **Weakness.**

**PSYCH** No flashing lights or hallucinations

**PAST MEDICAL HISTORY (PMI):**

**Past Illnesses**: **Ninguna, siempre he estado sano/a** (None, I’ve always been healthy.)

**Past surgeries**: None

**Pregnancy**: None

**Hospitalizations:** None

**Accidents/injuries: Nada importante, esguinces ocasionales en la infancia, etc.** (Nothing major, occasional childhood sprains, etc.)

**Immunizations: No recibo una vacuna anual contra la gripe y no recibí ninguna vacuna contra el COVID**. ( I do not get an annual flu shot and did not get any COVID vaccinations.)

*For [female] only:*

Menstrual history: N/A

------------------------------------------------------------------------------------------------------------

**OB/GYN:** N/A

**MEDICATIONS:**

Prescriptions: **Niguno** (None)

Over-the-counter drugs: **Tylenol para la fiebre y los dolores corporales.** <Si me lo piden si lo ayuda, **Creo que sí, un poquito.>** (Tylenol for the fever and body aches. If asked whether it helps, I think it helps a little.) You’ve been taking Tylenol according to the directions on the box (2 pills every 6-8 hours) since the fever began. The fever peaks at 102 and the Tylenol brings it down to 100.

Herbs: **Ninguna** (None)

Illicit/street drugs: **No, nunca.** (No, never)

Allergies [Drug/other): **Ninguna** (None)

**FAMILY MEDICAL HISTORY: Vengo de una familia muy sana. Trabajamos duro y jugamos duro hasta que morimos de vejez.** (You are not aware of any major health issues in your family. “I come from a very healthy family. We work hard and play hard until we die of old age.)

As the ages of the SPs portraying this case will vary, so will the ages and health status of their relatives. Please take time to fill in this portion with the ages and health status (either “Alive and Healthy” or “Deceased of Old Age”) of your imaginary family in keeping with your real age.

Father:

*age*

*Health status/history*

Mother:

*age*

*Health status/history*

Sibling(s):

*age*

*Health status/history*

Grandfather (paternal):

*age*

*Health status/history*

Grandmother (paternal):

*age*

*Health status/history*

Grandfather (maternal):

*age*

*Health status/history*

Grandmother (maternal)

*age*

*Health status/history*

**PRESENT LIVING SITUATION**

**Tengo una casa aquí en Las Vegas donde vivo con mi esposa/o. Tenemos dos hijos adultos.**

(You live in a house in Las Vegas with your spouse. Your 2 children are grown.)

If asked about sick contacts, i.e. Have you been around anyone who is sick? At home? At work?, you reply:

**No sé, no que yo sepa, pero es posible. Cuando veo a alguien con secreción nasal, estornudos, tos, generalmente me imagino que es la temporada de alergias. Ahora no estoy tan seguro/a…**

(Not that I’m aware of, but it’s possible. When I see someone with a runny nose, sneezing, coughing, I usually just figure it’s allergy season. Now I’m not so sure…)

**SOCIAL HISTORY:**

*Occupation:* **Soy dueño/a de un restaurante. Espero que no salgamos del negocio.** (I own a restaurant. I hope we don’t go out of business.)

*Marital Status:* **Casado/a** (Married)

*Support system:* **Tengo un gran sistema de apoyo de familia y amigos muy unidos que son como la familia.** (Large support system of close-knit family and friends who are like family.)

*Sleep pattern:* **No he tenido problemas para dormir últimamente. Siempre he dormido bien.**

(I haven’t had any trouble sleeping lately*.* I’ve always slept well, 6 -8 hours each night)

*Alcohol :* **3-4 tragos a la semana** (3-4 drinks a week)

*Tobacco :* **No, nunca.** (No, never)

*Diet :* **Como una dieta equilibrada en el restaurante y en casa. No hay nada más que excelentes cocineros en mi familia.** (I eat a balanced diet at the restaurant and at home. There are nothing but great cooks in my family.)

*Caffeine :* **1 taza cada mañana con el desayuno.** (1 cup each morning with breakfast)

*Exercise*: **Me cuido, salgo a caminar, y estoy de pie todo el día en el trabajo.** (I take care of myself, I take walks, and I’m on my feet all day at work.)

*Activities/hobbies :* **Paso el tiempo con mi familia. Jugando con mis nietos. Ir a los juegos de fútbol de mi familia.** Spending time with my family. Playing with my grandkids. Going to my family’s soccer games.

*Travel :* **Mi esposa/o y yo regresamos de España hace dos semanas.** (My husband/wife and I returned from Spain 2 weeks ago.)

*Sexual History:* **Soy activa/o con mi esposa/o.** (I’m active with my spouse.)

*Spirituality / Religion:* **Fui criado católico y crié a mis hijos católicos.** (I was raised Catholic and raised my kids Catholic.)

The two questions an SP can ask for this case are:

1. (Dx related) **¿Cuál crees que es el problema?** (What do you think the problem is?)
2. (Educational) **¿Cuándo puedo volver a trabajar?** (When can I go back to work?)

***Interpreter Information***

Beto/Berta Ruiz

TRAINING MATERIALS

**Reason for your today’s visit*-(¿Cuál es su razón para su cita hoy?)***

**When did you start having symptoms*-(¿Cuando empezaste a tener síntomas)***

**How many days have you felt like this*-(¿Cuantos dias has tenido esto?)***

**What is the frequency of your symptoms*-(¿Cuál es la frecuencia de sus síntomas?)***

**Please describe your symptoms*-(*** ***Por favor describa sus síntomas.)***

**Do you have a cough*-(Tiene tos?)***

**How frequently do you cough?*(¿Con qué frecuencia tose?)***

**Is it productive or dry*- (¿Está productiva o seca?)***

**Do you have a fever*-(¿Tiene fiebre?)***

**How high is your fever*- (¿Qué tan alta es tu fiebre?)***

**What is the pattern of your fever?** ***(¿Cuál es el patrón de tu fiebre?)***

**Did the Tylenol reduce your fever?** ***(¿El Tylenol redujo la fiebre?)***

**How much Tylenol are you taking?** ***(¿Cuánto Tylenol estás tomando?)***

**Do you have shortness of breath*- (¿Tiene dificultad de respirar?)***

**Can you breathe- *(¿Puede respirar?)***

**What is the severity or intensity of your pain? *- (¿Cuál es la gravedad o intensidad de sus síntomas?)***

**What are your aggravating or alleviating factors - *(¿Cuáles son los factores agravantes o atenuantes?)***

**Associated symptoms*- (¿Cuales son tus síntomas asociadas?)***

**How frequently are you having diarrhea?** ***(¿Con qué frecuencia tiene diarrea?)***

**Describe the diarrhea** ***(Describe la diarrhea.)***

**What is the consistency of it?** ***(¿Cuál es la consistencia de esto?)***

**Previous episodes*-(¿Ha tenido estos síntomas en el pasado?)***

**What worries you the most about this illness*-(¿Qué es lo que más se preocupa de esto?)***

**What effect does this have on your daily life? *- (¿Qué efecto tiene esto en su vida diaria?)***

**Past illnesses*- (¿Tiene alguna enfermedad pasada?)***

**Past surgeries- *(¿*** ***Ha tenido alguna cirugía anterior?)***

**Pregnancies? *- (¿Embarazos?)***

**Hospitalizations*-(¿Ha sido hospitalizado?)***

**Accidents/injuries*-(¿Ha tenido accidentes o lesiones?)***

**Immunizations*-(¿Sus vacunas están al día?)***

**Do you take medication-*(¿Toma medicamentos?)***

**Over-the-counter drugs*-(¿Toma medicamentos de venta libre?)***

**Herbs*-(¿Toma hierbas o suplementos?)***

**Illicit/street drugs*-(¿Usa drogas ilícitas?)***

**Do you have allergies*-(¿Tiene alergias o alergias a medicamentos?)***

**Are you allergic to any medications*-(¿Tiene alergias a medicamentos?)***

**What is your family medical history*-(¿Cuál es su historia médica familiar?)***

**In the past two weeks have you had contact with anyone who is sick? At home? At work? *- (¿Durante las dos semanas pasadas ha tenido contacto con alguien enfermo? ¿En casa? ¿O en el trabajo?)***

**Occupation*-(¿Cuál es su ocupación?)***

**Support system- *(¿Cual es us sistema de apoyo?)***

**Sleep pattern*-(¿Qué tan bien duerme por la noche?)***

**Alcohol*- (¿Bebe alcohol? ¿Con qué frecuencia?)***

**Tobacco*-(¿Fuma cigarillos o usa tabaco?)***

**Diet- *(¿Cómo es su dieta?)***

**Caffeine:**-***(¿Que es su consumo diario de cafeína?)***

**Exercise-*( ¿Hace ejercicio?)***

**Activities/hobbies-*(¿Cuáles son sus actividades y pasatiempos fuera del trabajo?)***

**Sexual History*-(¿Que es su historia sexual? ¿Está sexualmente activo/a?)***

**Spirituality */ Religion-(¿Cual es su religion?)***
