# Supplementary material for: Social Determinants of Health: A Multilingual Standardized Patient Case to Practice Interpreter Use in a Telehealth Visit
Source: MedEdPORTAL. 2023 Nov 14;19:11364. doi: 10.15766/mep_2374-8265.11364 (PMC10643468; doi:10.15766/mep_2374-8265.11364)
Supplement: Supplementary file 1 — SP Case - Spanish.docxSP Case - Tagalog.docxSP Case - Igbo.docxSP Case - French.docxSMI - Spanish.docxSMI - Tagalog.docxSMI - Igbo.docxSMI - French.docxSPL Rehearsal Script.docxDoor Instructions - Spanish and Tagalog.docxDoor Instructions - Igbo.docxDoor Instructions - French.docxFaculty Guide.pdfStudent Guide.pdfImportant Points Interpreters Telehealth.docxGraphic Instructional Tool.pdfSample Progress Note.docxProgress Note Grading Rubric.xlsx [file mep_2374-8265.11364-s001.zip › F. SMI - Tagalog.docx]

**Beto/Berta Ruiz – Tagalog Version**

***SP Educator information***

Presenting complaint: Extreme fatigue

Differential diagnoses: COVID-19, influenza, SARS-CoV-2

Patient demographics:

Age: Any

Sex: Any

Race: Asian and/or Hispanic

Height: Any

Weight: Any

Physical Findings the SP should NOT have (scars, etc. ): None

PROFILE

The patient is a non-English speaking man or woman who is complaining of extreme fatigue. The patient called the doctor’s office, was told to stay home and was given a telehealth appointment. The patient requires a translator during the appointment.

OBJECTIVES: history/physical/other:

- Develop ways to create an environment conducive to conducting a telehealth visit that includes an interpreter.
- Demonstrate appropriate history gathering and physical exam components while interviewing a patient with fatigue during a telehealth visit.
- Apply techniques from the interpreter services reference materials to interview a non-English language preference patient with an interpreter and critique a peer after observing.
- Integrate information from the case and faculty and peer feedback to create a progress note with an appropriate basic differential diagnosis and treatment plan for a patient with fatigue.

SPECIAL NEEDS/EQUIPMENT (over and above standard exam room set-up):

1. Computer devices with internet access (desktop computer with monitor, laptop computer, tablet, smartphone, etc.) for learner(s) and two standardized patients.
2. ZOOM, Google Hangouts, Webex, Skype, Facetime, or other online platform for telehealth meetings.

PURPOSE OF THIS ACTIVITY:

To practice interviewing a non-English language preference patient in a telehealth setting with an interpreter.

LAST USE OF THIS CASE:

April 22, 2021 for UNLVSOM Doctoring 2 week 13 (revised June 14, 2023)

**CASE AUTHORS:**

Gigi Guizado de Nathan, BA

Translation from the English by Marie Christine Padilla, BSHCA

PRESENTING SITUATION

and

INSTRUCTIONS TO THE STUDENT

Beto/Berta Ruiz

Beto/Berta Ruiz is an adult male or female who has been told to call TELEHEALTH SERVICES today for extreme fatigue. The patient does not speak English.

Vital signs:

T: 102° F oral Pulse: 75 bpm BP: 132/64 RR: 25

You are to:

- Develop ways to create an environment conducive to conducting a telehealth visit that includes an interpreter.
- Demonstrate appropriate history gathering and physical exam components while interviewing a patient with fatigue during a telehealth visit.
- Apply techniques from the interpreter services reference materials to interview a non-English language preference patient with an interpreter and critique a peer after observing.
- Integrate information from the case and faculty and peer feedback to create a progress note with an appropriate basic differential diagnosis and treatment plan for a patient with fatigue.

***SP Information***

Beto/Berta Ruiz

TRAINING MATERIALS

CASE SUMMARY

You are a non-English speaking person (use your own age and gender) who is complaining of extreme fatigue. Your bilingual spouse called the doctor’s office and was given a telehealth appointment. You need a translator for this appointment, as your spouse has gone to work, holding down the family restaurant.

You and your extended family own and operate restaurants in Las Vegas and North Las Vegas. Today is the third day in a row that you have stayed home with fatigue, fever, chills and cough. In the past 24 hours, diarrhea and runny nose have started, too. As a result, you’ve lost your appetite. You’ve had the flu before, but the severity of the fatigue is new to you. You have never felt so sick in your life.

Two weeks ago, you and your spouse returned from a dream vacation to Spain. Three days ago, the extreme fatigue set in while you were on a typical walk to the corner store. Fever, chills, and a dry cough soon followed.

You haven’t had much appetite since the runny nose started yesterday, along with the unsettled stomach and diarrhea.

Your spouse is concerned about your health. Deep down, you are too. Perhaps because it is easier on you emotionally and psychologically, you are remaining focused on keeping your business alive.

Your challenge, as the standardized patient, is:

1. To appropriately and accurately reveal the facts of the patient’s case through an interpreter in a telehealth setting.

PRESENTATION/EMOTIONAL TONE

When the student joins the video call you should be sitting in a chair wearing your regular clothes.

In general, Beto/Berta, and the translator, are pleasant and easy to talk to. You answer all questions directly without ‘dancing around the subject’. Beto/Berta will cough (into their elbow or a tissue) at the start of the encounter. S/He will also appear fatigued throughout the encounter.

OPENING LINE

In response to the typical opening question from the student, “What brings you in here today?” – you respond verbatim.

**“Ako ay pagod at nanghihina. Nahirapan nga akong maglakad pabalik galing sa tindahan. <umubo>”**

(I am so tired and weak. I barely made it back from my usual walk to the corner store. <cough>)

If invited to say more:

**“Nagduda nga ako kung makakalakad pa akong pabalik nang bahay para makabalik sa aking kama.** <pag tinanong, **mga 15 lakad kalayo>** (I really doubted I was gonna make it from my front door to my bed. < if asked, it’s 15 paces or so >)

If invited to say more:

**“Gusto ko na talagang makabalik sa trabaho.”** (I’d really like to get back to work.)

**HISTORY OF PRESENT ILLNESS (HPI)**:

*Onset:* **Ako ay nanghihina, mga tatlong araw na.**

*Duration:* **Tatlong araw.** 3 days

*Frequency:* **Palage.** Constant

*Quality/Description:* **Ako ay sobrang nanghihina, ni hindi ko kayang magbasa nang dyaryo habang nakahiga.** (I’m too weak to even read the newspaper while I’m in bed.)

*Severity/intensity:* **Sa buong buhay ko, ngayon lang ako nagkasakit nang ganito.** (I’ve never felt so sick in my life.)

*Location:* **Nananakit ang buong katawan ko.** (My whole body aches.)

*Aggravating /alleviating factors:* **Mas lalo akong nanghihina pag tumatayo ako, papuntang banyo. Walang nakakatulong sa panghihina kong ito.** (Getting up to go to the bathroom makes it worse. Nothing makes the fatigue better.)

*Associated symptoms:* **Lagnat, panlalamig, pananakit nang katawan, ubo, pagtatae, sipon.** (Fever, chills, body aches, cough, diarrhea, runny nose.) If asked, the constant fever ranges from 100 to 102, the cough is constant, the diarrhea is watery and happens about 4 times a day.

**RESPONSE DURING PHYSICAL EXAMINATION:** (ROM, pain, procedure responses during PE to make case clinically accurate): N/A, There is no physical examination during this case.

**Your chief concern / patient perspective of illness** (If the student asks, “What concerns you most about this?” (Or something of that nature), you reply that:

**Gustong gusto ko nang bumalik sa trabaho.**

(I want to get back to work as soon as possible.)

If the student asks, “what effect does this have on your daily life?**” (**Or something of that nature, you reply that:

**Hindi ako makapagtrabaho. Lahat nang kabuhayan ko, nang pamilya ko, ng mga empleyado ko ay nakasalalay sa akin.** (I can’t go to work. The future of my business, and the livelihoods of my family and employees are all depending on me.)

**REVIEW OF SYSTEMS** (Items in **bold** indicate a “yes” response)

**GENERAL** – No tearing or redness of the eyes noted. **Body aches.**

**Head** – No headache

**ENT** – No tinnitus (ringing in ears), no loss of hearing. No sensitivity to noises. **Runny nose.**

**EYES:** No loss of vision, no light sensitivity**.** Past examination (in the last year) was normal.

**CV**- no chest pain, no palpitations

**LUNG** –No hemoptysis or wheezing. **Dry cough**.

**Genitourinary** – No problems with urination. No blood in the urine. Male: No erectile dysfunction. Female:(see menstrual history below**).**

**GI** – no abdominal pain. No problems with bowels, no constipation, no nausea. **Diarrhea and loss of appetite.**

**MUSCULOSKELETAL** – No joint pain or muscle pain /spasm.

**ENDOCRINE**- No hot flashes, hair loss or temperature sensitivity, no increased thirst, no recent weight loss. **Chills and** **fever**.

**SKIN**: No new rashes or other problems

**NEUROLOGIC**: No numbness, tingling, tremor, fainting, memory loss or loss of balance. **Weakness.**

**PSYCH** No flashing lights or hallucinations

**PAST MEDICAL HISTORY (PMI):**

**Past Illnesses**: **Wala. Noon paman, di ako nagkakasakit.** (None, I’ve always been healthy.)

**Past surgeries**: None

**Pregnancy**: None

**Hospitalizations:** None

**Accidents/injuries: Wala naman masyado, siguro mga sugat bata lang noon, etc.** (Nothing major, occasional childhood sprains, etc.)

**Immunizations: Hindi ako nakakakuha ng taunang bakuna laban sa trangkaso at hindi ako nakakuha ng anumang pagbabakuna sa COVID.** (I do not get an annual flu shot and did not get any COVID vaccinations.)

*For [female] only:*

Menstrual history: N/A

------------------------------------------------------------------------------------------------------------

**OB/GYN:** N/A

**MEDICATIONS:**

Prescriptions: **Wala** (None)

Over-the-counter drugs: **Tylenol lang para sa paglalagnat at pananakit nang katawan.** <Kapag tinanong, **Sa aking palagay, ito ay nakakatulong nang konti, kahit papano.>** (Tylenol for the fever and body aches. If asked whether it helps, I think it helps a little.) You’ve been taking Tylenol according to the directions on the box (2 pills every 6-8 hours) since the fever began. The fever peaks at 102 and the Tylenol brings it down to 100.

Herbs: **Wala** (None)

Illicit/street drugs: **Hindi, kahit kailan.** (No, never)

Allergies [Drug/other): **Wala** (None)

**FAMILY MEDICAL HISTORY: Sa aking pagkakaalam, wala sa aking pamilya ang may malubhang sakit. Kami ay malusog. Lahat kami ay masipag at nag-e-enjoy lang sa buhay hanggang sa mamatay na sa pagtanda.** (You are not aware of any major health issues in your family. “I come from a very healthy family. We work hard and play hard until we die of old age.)

As the ages of the SPs portraying this case will vary, so will the ages and health status of their relatives. Please take time to fill in this portion with the ages and health status (either “Alive and Healthy” or “Deceased of Old Age”) of your imaginary family in keeping with your real age.

Father:

*age*

*Health status/history*

Mother:

*age*

*Health status/history*

Sibling(s):

*age*

*Health status/history*

Grandfather (paternal):

*age*

*Health status/history*

Grandmother (paternal):

*age*

*Health status/history*

Grandfather (maternal):

*age*

*Health status/history*

Grandmother (maternal)

*age*

*Health status/history*

**PRESENT LIVING SITUATION**

**Ako ay nakatira sa isang bahay sa Las Vegas. Kasama ko ang aking asawa at dalawang anak na nasa tamang edad na.**

(You live in a house in Las Vegas with your spouse. Your 2 children are grown.)

If asked about sick contacts, i.e. Have you been around anyone who is sick? At home? At work?, you reply:

**Sa pagkakaalam ko, di ako nakipaghalubilo sa taong may sakit, subalit ito ay posible. Dahil, kapag may nakikita akong taong may sipon, humahaching, umuubo, inakala kong may allergy lang sila. Ngayon, hindi na ako sigurado…**

(Not that I’m aware of, but it’s possible. When I see someone with a runny nose, sneezing, coughing, I usually just figure it’s allergy season. Now I’m not so sure…)

**SOCIAL HISTORY:**

*Occupation:* **Ako ay isang restaurant owner. Sana di ako mawalan nang panghanapbuhay.** (I own a restaurant. I hope we don’t go out of business.)

*Marital Status:* **Kasal** (Married)

*Support system:* **Malaki ang aking support system. Mula sa aking mga malapit na pamilya at kamag-anak hanggang sa aking mga kaibigan na tinuturing ko narin na pamilya.** (Large support system of close-knit family and friends who are like family.)

*Sleep pattern:* **Nakatulog ako nang mahimbing. Palagi naman akong natutulog nang mga anim hanggang walong tulog bawat gabi.**

(I haven’t had any trouble sleeping lately*.* I’ve always slept well, 6 -8 hours each night)

*Alcohol :* **tatlo hanggang apat na inom bawat lingo** (3-4 drinks a week)

*Tobacco :* **Hindi, kahit kailan.** (No, never)

*Diet :* **Ako ay kumakain nang masustansya at balanceng diyeta. Lahat sa aking pamilya ay magaling magluto.** (I eat a balanced diet at the restaurant and at home. There are nothing but great cooks in my family.)

*Caffeine :* **Isang tasa bawat umaga, pang agahan.** (1 cup each morning with breakfast)

*Exercise*: **Iniingatan ko ang aking kalusugan, lagi akong nag-la-lakad, at palagi akong gumagalaw habang nasa trabaho.** (I take care of myself, I take walks, and I’m on my feet all day at work.)

*Activities/hobbies :* **Mahilig akong mag-spend time sa aking pamilya. Nakikipaglaro sa aking mga apo. Nag-a-attend nang soccer games nang aking pamilya.** Spending time with my family. Playing with my grandkids. Going to my family’s soccer games.

*Travel :*  **Ako at nang asawa ko ay kababalik palamang nang Spain mga dalawang lingo na.** (My husband/wife and I returned from Spain 2 weeks ago.)

*Sexual History:* **Ako at nang asawa ko ay actibo sa pagtatalik.** (I’m active with my spouse.)

*Spirituality / Religion:* **Ako at ang aking mga anak ay pinalaking Catolico.** (I was raised Catholic and raised my kids Catholic.)

The two questions an SP can ask for this case are:

1. (Dx related) **¿Sa iyong palagay, ano ang problema?** (What do you think the problem is?)
2. (Educational) **¿Kailan ako makakabalik nang trabaho?** (When can I go back to work?)

***Interpreter Information***

Beto/Berta Ruiz

TRAINING MATERIALS

**Reason for your today’s visit*-(¿Ano ang rason sa iyong pag-bisita ngayon?)***

**When did you start having symptoms*-(¿Kailan nag umpisa ang iyong sintomas)***

**How many days have you felt like this*-(¿Gaano muna katagal nararamdaman ito?)***

**What is the frequency of your symptoms*-(¿Gaano kadalas mong nararamdaman ang sintomas mo?)***

**Please describe your symptoms*-(*** ***Maaari mu bang i-describe ang iyong mga sintomas.)***

**Do you have a cough*-(May ubo ka ba?)***

**How frequently do you cough?**-***(Gaano ka kadalas umubo?)***

**Is it productive or dry*- (¿Ito ba ay madami o ito ba ay tuyot?)***

**Do you have a fever*-(¿May lagnat ka ba?)***

**How high is your fever*- (¿Gaano kataas ang iyong lagnat?)***

**What is the pattern of your fever?**-***(Anong klase ang iyong lagnat?)***

**Did the Tylenol reduce your fever?**-***(Uminum ka ba nang Tyleno para sa iyong lagnat?)***

**How much Tylenol are you taking?**-***(Gaano karame ang ininum mong Tylenol?)***

**Do you have shortness of breath*- (¿Nahihirapan ka bang huminga?)***

**Can you breathe- *(¿Nakakahinga ka ba?)***

**What is the severity or intensity of your pain? *- (¿Gaano kalala or kalakas ang iyong pananakit?)***

**What are your aggravating or alleviating factors - *(¿Ano ang nakakaargabyado o nakakatulong sa iyong pakiramdam?)***

**Associated symptoms*- (¿May iba ka pa bang sintomas?)***

**How frequently are you having diarrhea?**-***(Gaano kadalas and iyong diarea/pagtatae?)***

**Describe the diarrhea**-***(Maari mu bang i-describe ang iyong diarea/pagtatae?)***

**What is the consistency of it?**-***(Ano ang itsura nang iyong diarea/pagtatae?)***

**Previous episodes*-(¿Naramdaman mo na ba itong sintomas na ito dati?)***

**What worries you the most about this illness*-(¿Ano ang ikinababahala mo sa sakit na ito?)***

**What effect does this have on your daily life? *- (¿Ano ang ipekto nito sa pang-araw araw mong buhay?)***

**Past illnesses*- (¿Mayroon ka bang dating karamdaman?)***

**Past surgeries- *(¿*** ***Dating operasyon?)***

**Pregnancies? *- (¿Pagbubuntis?)***

**Hospitalizations*-(¿Na ospital ka na ba?)***

**Accidents/injuries*-(¿ Dating aksidente/pinsala?)***

**Immunizations*-(¿Mga bakuna?)***

**Do you take medication-*(¿Ikaw ba ay umiinom nang gamot?)***

**Over-the-counter drugs*-(¿Counter na gamot?)***

**Herbs*-(¿Halamang gamot?)***

**Illicit/street drugs*-(¿Bawal na gamot?)***

**Do you have allergies*-(¿May allergies ka ba?)***

**Are you allergic to any medications*-(¿May allergic reaction ka ba sa kahit anong gamot?)***

**What is your family medical history*-(¿Ano ang medical istorya nang iyong pamilya?)***

**In the past two weeks have you had contact with anyone who is sick? At home? At work? *- (¿Sa nakaraang dalawang lingo, nakahalubilo mo ba ang taong may sakit? Sa bahay? Trabaho?)***

**Occupation*-(¿Ano ang trabaho mo?)***

**Support system- *(¿May support system ka ba?)***

**Sleep pattern*-(¿Paano ang tulog mo?)***

**Alcohol*- (¿Umiinon ka ba nang alak?)***

**Tobacco*-(¿Naninigarilyo ka ba?)***

**Diet- *(¿Nag-di-diyeta ka ba?)***

**Caffeine:**-***(¿Uiimum ka ba kang inuming may kapeina?)***

**Exercise-*( ¿Nag-ehersisyo ka ba?)***

**Activities/hobbies-*(¿Ano ang mahilig mong gawin o libangan mo?)***

**Sexual History*-(¿Ano ang iyong sekswal na kasaysayan?)***

**Spirituality */ Religion-(¿Ano ang iyong paniniwala/relihiyon?)***

**Test (Medical Test)-*(¿Medikal na pagsusuri?)***

**Chronic*-(¿Malubha?)***

**Wheezing*-(¿Humihika?)***

**Palpitation*-(¿Irregular na pag-tibok nang puso?)***

**Allergy*-(¿Allergy?)***

**Social (Gathering)*-(¿Salu-Salo?)***

**Infection*-(¿Impeksiyon?)***

**Anemic*-(¿Anemik?)***

**Hyperthyroidism*-(¿Hyperthyroidism?)***

**Metabolic Rate*-(¿Metabolic Rate?)***

**Thyroid*-(¿Thyroid?)***

**Check (to check what is known)*-(¿Tiyakin ex. check that your temp is less than 100 deg Fahrenheit)***

**Check (to check what is not known)*-(¿Tingnan ex. check your temperature)***

**Sanitize*-(¿Nag-sa-sanitize ka?)***

**Self-Isolate*-(¿Self-Isolate/Pag-bu-bukod sa sarili?)***
