# Supplementary material for: Social Determinants of Health: A Multilingual Standardized Patient Case to Practice Interpreter Use in a Telehealth Visit
Source: MedEdPORTAL. 2023 Nov 14;19:11364. doi: 10.15766/mep_2374-8265.11364 (PMC10643468; doi:10.15766/mep_2374-8265.11364)
Supplement: Supplementary file 1 — SP Case - Spanish.docxSP Case - Tagalog.docxSP Case - Igbo.docxSP Case - French.docxSMI - Spanish.docxSMI - Tagalog.docxSMI - Igbo.docxSMI - French.docxSPL Rehearsal Script.docxDoor Instructions - Spanish and Tagalog.docxDoor Instructions - Igbo.docxDoor Instructions - French.docxFaculty Guide.pdfStudent Guide.pdfImportant Points Interpreters Telehealth.docxGraphic Instructional Tool.pdfSample Progress Note.docxProgress Note Grading Rubric.xlsx [file mep_2374-8265.11364-s001.zip › G. SMI - Igbo.docx]

**Ogo Azunna – Igbo Version**

***SP Educator information***

Presenting complaint: Extreme fatigue

Differential diagnoses: COVID-19, influenza, SARS-CoV-2

Patient demographics:

Age: Any

Sex: Any

Race: Black

Height: Any

Weight: Any

Physical Findings the SP should NOT have (scars, etc. ): None

PROFILE

The patient is a non-English speaking man or woman who is complaining of extreme fatigue. The patient called the doctor’s office, was told to stay home and was given a telehealth appointment. The patient requires a translator during the appointment.

OBJECTIVES: history/physical/other:

- Develop ways to create an environment conducive to conducting a telehealth visit that includes an interpreter.
- Demonstrate appropriate history gathering and physical exam components while interviewing a patient with fatigue during a telehealth visit.
- Apply techniques from the interpreter services reference materials to interview a non-English language preference patient with an interpreter and critique a peer after observing.
- Integrate information from the case and faculty and peer feedback to create a progress note with an appropriate basic differential diagnosis and treatment plan for a patient with fatigue.

SPECIAL NEEDS/EQUIPMENT (over and above standard exam room set-up):

1. Computer devices with internet access (desktop computer with monitor, laptop computer, tablet, smartphone, etc.) for learner(s) and two standardized patients.
2. ZOOM, Google Hangouts, Webex, Skype, Facetime, or other online platform for telehealth meetings.

PURPOSE OF THIS ACTIVITY:

To practice interviewing a non-English language preference patient in a telehealth setting with an interpreter.

LAST USE OF THIS CASE: (revised June 14, 2023)

**CASE AUTHORS:**

Gigi Guizado de Nathan, BA

Translation from the English by Ogonnaya Onyema DNP, RN, CMSRN

PRESENTING SITUATION

and

INSTRUCTIONS TO THE STUDENT

Ogo Azunna

Ogo Azunna is an adult male or female who has been told to call TELEHEALTH SERVICES today for extreme fatigue. The patient does not speak English.

Vital signs:

T: 102° F oral Pulse: 75 bpm BP: 132/64 RR: 25

You are to:

- Develop ways to create an environment conducive to conducting a telehealth visit that includes an interpreter.
- Demonstrate appropriate history gathering and physical exam components while interviewing a patient with fatigue during a telehealth visit.
- Apply techniques from the interpreter services reference materials to interview a non-English language preference patient with an interpreter and critique a peer after observing.
- Integrate information from the case and faculty and peer feedback to create a progress note with an appropriate basic differential diagnosis and treatment plan for a patient with fatigue.

***SP Information***

Ogo Azunna

TRAINING MATERIALS

CASE SUMMARY

You are a non-English speaking person (use your own age and gender) who is complaining of extreme fatigue. Your bilingual spouse called the doctor’s office and was given a telehealth appointment. You need a translator for this appointment, as your spouse has gone to work, holding down the family restaurant.

You and your extended family own and operate West African restaurants in Las Vegas and North Las Vegas. Today is the third day in a row that you have stayed home with fatigue, fever, chills and cough. In the past 24 hours, diarrhea and runny nose have started, too. As a result, you’ve lost your appetite. You’ve had the flu before, but the severity of the fatigue is new to you. You have never felt so sick in your life.

Two weeks ago, you and your spouse returned from a dream vacation to Spain. Three days ago, the extreme fatigue set in while you were on a typical walk to the corner store. Fever, chills, and a dry cough soon followed.

You haven’t had much appetite since the runny nose started yesterday, along with the unsettled stomach and diarrhea.

Your spouse is concerned about your health. Deep down, you are too. Perhaps because it is easier on you emotionally and psychologically, you are remaining focused on keeping your business alive.

Your challenge, as the standardized patient, is:

1. To appropriately and accurately reveal the facts of the patient’s case through an interpreter in a telehealth setting.

PRESENTATION/EMOTIONAL TONE

When the student joins the video call you should be sitting in a chair wearing your regular clothes.

In general, Ogo, and the translator, are pleasant and easy to talk to. You answer all questions directly without ‘dancing around the subject’. Ogo will cough (into their elbow or a tissue) at the start of the encounter. S/He will also appear fatigued throughout the encounter.

OPENING LINE

In response to the typical opening question from the student, “What brings you in here today?” – you respond verbatim.

**“Ike gwụrụ m. enweghị m ike ịlachite mgbe m gara ebe ahịa** <ụkwara>

(I am so tired and weak. I barely made it back from my usual walk to the corner store. <cough>)

If invited to say more:

**“Enwere m obi abụọ na-agaghi m enwe ike isi na-ịhụ ụlọm rute na-akwa m. < ọ abụrụ na ajụrụ gị, ọ dị 15 paces ma ọ bụ ya.>** (I really doubted I was gonna make it from my front door to my bed. < if asked, it’s 15 paces or so >)

If invited to say more:

**“Oga amasim ilaghachi n’oru.”** (I’d really like to get back to work.)

**HISTORY OF PRESENT ILLNESS (HPI)**:

*Onset:* **Ike ogwugwu bidoro abali ato, garaaga.**  (The tiredness started 3 days ago)

*Duration:*  **Abali ato. (**3 days)

*Frequency:* O**genile.** (Constant)

*Quality/Description:* **Adighị m ike ọbụla ịgụ akwukwo akuko mgbe m dinara.** (I’m too weak to even read the newspaper while I’m in bed.)

*Severity/intensity:* **Enwetụbeghị m ọrịa otúa na-ahum na-ndụ m.** (I’ve never felt so sick in my life.)

*Location:* **Ahụm niile na egbummgbu.** (My whole body aches.)

*Aggravating /alleviating factors:* **Ibili na-aga ime ụlọ ịwụ ahụ na-eme ka ọ ka njọ. Ọ dịghị ihe na-eme ka ike ọgwugwu ka mma..** (Getting up to go to the bathroom makes it worse. Nothing makes the fatigue better.)

*Associated symptoms:* A**huọkụ, akpata oyi, ahụ mgbu, ukwara, afọ osisa, imi na-agba agba.** (Fever, chills, body aches, cough, diarrhea, runny nose.) If asked, the constant fever ranges from 100 to 102, the cough is constant, the diarrhea is watery and happens about 4 times a day.

**RESPONSE DURING PHYSICAL EXAMINATION:** (ROM, pain, procedure responses during PE to make case clinically accurate): N/A, There is no physical examination during this case.

**Your chief concern / patient perspective of illness** (If the student asks, “What concerns you most about this?” (Or something of that nature), you reply that:

**Achọrọm ịlaghachi n’ọrụ ọsịsọ.** (I want to get back to work as soon as possible.)

If the student asks, “what effect does this have on your daily life?**” (**Or something of that nature, you reply that:

**Enweghị m ike ịga ọrụ, ọdịnịhu nke azụm ahịa m, yana odimma ezinaụlọ m na-di ọrụ m niile dabere na m.** (I can’t go to work. The future of my business, and the livelihoods of my family and employees are all depending on me.)

**REVIEW OF SYSTEMS** (Items in **bold** indicate a “yes” response)

**GENERAL** – No tearing or redness of the eyes noted. **Body aches.**

**Head** – No headache

**ENT** – No tinnitus (ringing in ears), no loss of hearing. No sensitivity to noises. **Runny nose.**

**EYES:** No loss of vision, no light sensitivity**.** Past examination (in the last year) was normal.

**CV**- no chest pain, no palpitations

**LUNG** –No hemoptysis or wheezing. **Dry cough**.

**Genitourinary** – No problems with urination. No blood in the urine. Male: No erectile dysfunction. Female:(see menstrual history below**).**

**GI** – no abdominal pain. No problems with bowels, no constipation, no nausea. **Diarrhea and loss of appetite.**

**MUSCULOSKELETAL** – No joint pain or muscle pain /spasm.

**ENDOCRINE**- No hot flashes, hair loss or temperature sensitivity, no increased thirst, no recent weight loss. **Chills and** **fever**.

**SKIN**: No new rashes or other problems

**NEUROLOGIC**: No numbness, tingling, tremor, fainting, memory loss or loss of balance. **Weakness.**

**PSYCH** No flashing lights or hallucinations

**PAST MEDICAL HISTORY (PMI):**

**Past Illnesses**: **Ọ dịghi. Ahụ gbasiri m ike mgbe niile.** (None, I’ve always been healthy.)

**Past surgeries**: ọ dighị (None)

**Pregnancy**: ọ dighị (None)

**Hospitalizations:** ọ dighị (None)

**Accidents/injuries: Ọ dighị ihe dị mkpa, nmeru ahu nwata oge ụfọdụ, etc.** (Nothing major, occasional childhood sprains, etc.)

**Immunizations: Anaghị m ịgba ọgwụ mgbochi afọ ọ bụla ma enwetaghị m ọgwụ mgbochi COVID ọ bụla.**

(I do not get an annual flu shot and did not get any COVID vaccinations.)

*For [female] only:*

Menstrual history: N/A

------------------------------------------------------------------------------------------------------------

**OB/GYN:** N/A

**MEDICATIONS:**

Prescriptions: **Ọ dịghi** (None)

Over-the-counter drugs: **Tylenol maka ahụ ọkụ na ahụ mgbụ. <ọ bụrụ na a jụọ ya ma ọ na-enyere aka. Echere m na ọ na-enyere aka ntakịrị>** (Tylenol for the fever and body aches. If asked whether it helps, I think it helps a little.) You’ve been taking Tylenol according to the directions on the box (2 pills every 6-8 hours) since the fever began. The fever peaks at 102 and the Tylenol brings it down to 100.

Herbs: **Ọ dighị** (None)

Illicit/street drugs: **Mba, emeghi.** (No, never)

Allergies [Drug/other): **Ọ dighị.** (None)

**FAMILY MEDICAL HISTORY: Ị maghị banyere nsogbu ahuike ọ bụla na ezinụlọgị. esim. ezinụlọ gbasirị ike. anyị na-arụsi ọrụ ike ma na-egwu egwu ruo mgbe anyi nwụrụ na-agadi.** (You are not aware of any major health issues in your family. “I come from a very healthy family. We work hard and play hard until we die of old age.)

As the ages of the SPs portraying this case will vary, so will the ages and health status of their relatives. Please take time to fill in this portion with the ages and health status (either “Alive and Healthy” or “Deceased of Old Age”) of your imaginary family in keeping with your real age.

Father:

*age*

*Health status/history*

Mother:

*age*

*Health status/history*

Sibling(s):

*age*

*Health status/history*

Grandfather (paternal):

*age*

*Health status/history*

Grandmother (paternal):

*age*

*Health status/history*

Grandfather (maternal):

*age*

*Health status/history*

Grandmother (maternal)

*age*

*Health status/history*

**PRESENT LIVING SITUATION**

**Gi na nwunye gi obi n’ ulo di na Las vegas. ụnụ gị abụọ étochala.**

(You live in a house in Las Vegas with your spouse. Your 2 children are grown.)

If asked about sick contacts, i.e. Have you been around anyone who is sick? At home? At work?, you reply:

**Ọbụghị na m matara maka ya, mana enwere ike. Mgbe m huru onye nwere imi na-agba agba, izeuzere, ukwara, Ana m ahu karịya na ọ bụ oge nfụkasị ahụ. Ugbu a ejighịm aka**

(Not that I’m aware of, but it’s possible. When I see someone with a runny nose, sneezing, coughing, I usually just figure it’s allergy season. Now I’m not so sure…)

**SOCIAL HISTORY:**

*Occupation:* **Enwere m ụlọ oriri. Enwere m olile anya na anyị agaghị apụ ahịa.** (I own a restaurant. I hope we don’t go out of business.)

*Marital Status:* **keolulu** (Married)

*Support system:* **Enwere m mnukwu nkwado ebe ezinụlọ m nọ na ndienyim.** (Large support system of close-knit family and friends who are like family.)

*Sleep pattern:* **Enwebeghi m nsogbu ọ bụla ịrahu ụra n’oge na-adịbeghi anya. Anọ m na-ehi ụra nke ọma mgbe niile.** (I haven’t had any trouble sleeping lately*.* I’ve always slept well, 6 -8 hours each night)

*Alcohol :* U**gboro atọ ma ọbụ anọ nime ọtụ izu ụka** (3-4 drinks a week)

*Tobacco :* M**ba, emeghi.** (No, never)

*Diet :* M**na eri ezigbo nri na ụlọ oriri yana ụlọ m. ọdighị ihe dị ka ezigbo nri na ezina ụlọ m.** (I eat a balanced diet at the restaurant and at home. There are nothing but great cooks in my family.)

*Caffeine :* Otu **iko ụtụtụ ọbụla naṅri.** (1 cup each morning with breakfast)

*Exercise*: **Anam elekota onwem, anàmagari ije, anàmano nuakwum ege niile mno nọrụ.** (I take care of myself, I take walks, and I’m on my feet all day at work.)

*Activities/hobbies :* **Ana m anodu ọnọdu na ezinụlọ, mmụ na ụmụ ụmụ m na-egwuriegwu, ana m aga egwuri egwu ezinụlọm.** Spending time with my family. Playing with my grandkids. Going to my family’s soccer games.

*Travel :* **Mụ na dim/nwunyem si Spain lata izu ụka abụọ garaaga.** (My husband/wife and I returned from Spain 2 weeks ago.)

*Sexual History:* M**ụ na nwunyem na-arụsi ọrụ ike** (I’m active with my spouse.)

*Spirituality / Religion:* **Azurum na okpukpe katolik, azukwaram ụmụm na okpukpe katolik.** (I was raised Catholic and raised my kids Catholic.)

The two questions an SP can ask for this case are:

1. (Dx related) G**ini ka ị chere nsogbua bụ?** (What do you think the problem is?)
2. (Educational) **Kedu mgbem ga alaghachi n’ọrụ?** (¿When can I go back to work?)

***Interpreter Information***

Ogo Azunna

TRAINING MATERIALS

**Reason for your today’s visit*-(Gini mere iji bia n’ubochi taa?)***

**When did you start having symptoms*-(Kedụ mgbe ị malitere inwe ihe mgbaàmà?)***

**How many days have you felt like this*-(Ụbọchi olé ka ọdị gị otú a?)***

**What is the frequency of your symptoms*-( Ugboro olé ka ina enwe ihe mgbàamà?)***

**Please describe your symptoms*-(*** ***Biko kọwaa ihe mgbaàmà gị)***

**Do you have a cough*-(Ị nwere ụkwara?)***

**How frequently do you cough?*(Ugboro olé ka ị na akwa ụkwara?)***

**Is it productive or dry*- (Ị na-akwaputa ihe ka okpọrọ ṅkụ?)***

**Do you have a fever*-((Ị nwere ahụ ọkụ?)***

**How high is your fever*- (Kedụ ka ahụ ọkụ gị dị?)***

**What is the pattern of your fever?** ***(Kedụ usoro ahụ ọkụ gị ?)***

**Did the Tylenol reduce your fever?** ***(Tylenol oweturu ahu oku gị?)***

**How much Tylenol are you taking?** ***(Tylenol olé ka ịna ańuu?)***

**Do you have shortness of breath?*- (Ị nwere ụme di mkpụ mkpụ?)***

**Can you breathe?- *(Ị nwere ike iku ụme?)***

**What is the severity or intensity of your pain? *- (Kedu ka ike nke ahụ mgbu gi dị?)***

**What are your aggravating or alleviating factors? - *(Kedu ihe bụ ihe na-eme ka ọ ka njọ ma ọ bụ belata?)***

**Do you have any associated symptoms*- (Ị nwere ihe mgbaàmà ọ bụla metụtara nka?)***

**How frequently are you having diarrhea?** **(Ugboro olé ka ị na-aria afọ osịsa?)**

**Describe the diarrhea** ***(Kọwaa afọ ọsịsa gị)***

**What is the consistency of it?** ***(Kedu ka agbanwe agbanwe ya dị?)***

**Have you had these symptoms before?*-(Ị nweela mgbaàmà ndi a mbụ?)***

**What worries you the most about this illness?*-(Kedu ihe kacha echegbu gi gbasara oria a?)***

**What effect does this have on your daily life? *- (Kedu mmetuta ihe ndia nwere na n’dụ gi kwa ubọchị?)***

**What past illnesses have you had?*- (Kedu ọrịa ndị gara aga ị nwere?)***

**Have you had past surgeries?- *(Ị nwere ịwa ahụ n’oge gara aga?)***

**Pregnancies? *- (Afọime?)***

**Have you been hospitalized?*-(Ị nọ la n’ ụlọ ọgwụ?)***

**Have you had accidents/injuries?*-(Ị nwee la ihe mberede/maọbụ mmerụahụ?)***

**Are your immunizations up to date?*-(Ị gbala ọgwụ mgbochi gị ruo ụbọchi?)***

**Do you take medication?-*(Ị na-anu ọgwụ?)***

**Do you take any over-the-counter drugs?*-(Ị na-aṅụ ọgwụ ṅdi kemist?)***

**Do you take herbs or supplements?*-(Ị na-aṅụ ọgwụ igbo ma ọ bu ọgwụ mgbakwunye?)***

**Do you use illicit/street drugs?*-(Ị na-aṅụ ọgwụ ọjọọ?)***

**Do you have allergies?*-(Ị nwere ihe nfụkasị ahụ?)***

**Are you allergic to any medications?*-(Onwere ọgwụ ọ bula na-eme gị ihe nfụkasi ahụ?)***

**What is your family medical history?*-(Kedu akụkọ gbasara ọgwụ na ahu ike ezinaụlọ gị?)***

**In the past two weeks have you had contact with anyone who is sick? At home? At work? *- (Na-ime izu uka abuo garaaga inmena mmeko gina onye oria? na ụlọ gị? na ụlọ ọrụ gị?)***

**What is your occupation*-(Gini bu ọrụ aka gị?)***

**What is your support system- *(Kedu usoro nkwado gi?)***

**Do you sleep well?*-(Ị na-arahụ ụra uṟa nke ọma?)***

**Do you drink alcohol? How often?*- (Ị na-aṅụ mmanya na egbuegbu, kedu ugboro olé?)***

**Do you smoke or use tobacco?*-(Ị na-ese anwụrụ maọbụ ịna-eji utaba?)***

**How is your diet?- *(Kedu ka iri nri gi si di?)***

**How much caffeine do you drink?**-***( Ị na ańụ kafine olé ?)***

**Do you exercise?-*(Ị na-eme mmegari ahụ?)***

**What are your activities/hobbies-*(Kedu ihe omume gị ma-ọbụ ihe omume ntụrụndụ gị?)***

**Are you sexually active? What is your sexual history?*-(Ị na-enwe mmekọahụ? Ginibu akuka mmekọahu gị?)***

**What is your Spirituality */* Religion*? -(Gini bu okpukpe gi?)***
