# Supplementary material for: Social Determinants of Health: A Multilingual Standardized Patient Case to Practice Interpreter Use in a Telehealth Visit
Source: MedEdPORTAL. 2023 Nov 14;19:11364. doi: 10.15766/mep_2374-8265.11364 (PMC10643468; doi:10.15766/mep_2374-8265.11364)
Supplement: Supplementary file 1 — SP Case - Spanish.docxSP Case - Tagalog.docxSP Case - Igbo.docxSP Case - French.docxSMI - Spanish.docxSMI - Tagalog.docxSMI - Igbo.docxSMI - French.docxSPL Rehearsal Script.docxDoor Instructions - Spanish and Tagalog.docxDoor Instructions - Igbo.docxDoor Instructions - French.docxFaculty Guide.pdfStudent Guide.pdfImportant Points Interpreters Telehealth.docxGraphic Instructional Tool.pdfSample Progress Note.docxProgress Note Grading Rubric.xlsx [file mep_2374-8265.11364-s001.zip › H. SMI - French.docx]

**Bertrand/Berthe Rousseau – French Version**

***SP Educator information***

Presenting complaint: Extreme fatigue

Differential diagnoses: COVID-19, influenza, SARS-CoV-2

Patient demographics:

Age: Any

Sex: Any

Race: Any

Height: Any

Weight: Any

Physical Findings the SP should NOT have (scars, etc.): None

PROFILE

The patient is a non-English speaking man or woman who is complaining of extreme fatigue. The patient called the doctor’s office, was told to stay home and was given a telehealth appointment. The patient requires a translator during the appointment.

OBJECTIVES: history/physical/other:

- Develop ways to create an environment conducive to conducting a telehealth visit that includes an interpreter.
- Demonstrate appropriate history gathering and physical exam components while interviewing a patient with fatigue during a telehealth visit.
- Apply techniques from the interpreter services reference materials to interview a non-English language preference patient with an interpreter and critique a peer after observing.
- Integrate information from the case and faculty and peer feedback to create a progress note with an appropriate basic differential diagnosis and treatment plan for a patient with fatigue.

SPECIAL NEEDS/EQUIPMENT (over and above standard exam room set-up):

1. Computer devices with internet access (desktop computer with monitor, laptop computer, tablet, smartphone, etc.) for learner(s) and two standardized patients.
2. ZOOM, Google Hangouts, Webex, Skype, Facetime, or other online platform for telehealth meetings.

PURPOSE OF THIS ACTIVITY:

To practice interviewing a non-English language preference patient in a telehealth setting with an interpreter.

LAST USE OF THIS CASE:

April 22, 2021 for UNLVSOM Doctoring 2 week 13 (revised June 14, 2023)

**CASE AUTHORS:**

Gigi Guizado de Nathan, BA

Translation from the English by Steven Fehr, MFA

PRESENTING SITUATION

and

INSTRUCTIONS TO THE STUDENT

Bertrand/Berthe Rousseau

Bertrand/Berthe Rousseau is an adult male or female who has been told to call TELEHEALTH SERVICES today for extreme fatigue. The patient does not speak English.

Vital signs:

T: 102° F oral Pulse: 75 bpm BP: 132/64 RR: 25

You are to:

- Develop ways to create an environment conducive to conducting a telehealth visit that includes an interpreter.
- Demonstrate appropriate history gathering and physical exam components while interviewing a patient with fatigue during a telehealth visit.
- Apply techniques from the interpreter services reference materials to interview a non-English language preference patient with an interpreter and critique a peer after observing.
- Integrate information from the case and faculty and peer feedback to create a progress note with an appropriate basic differential diagnosis and treatment plan for a patient with fatigue.

***SP Information***

Bertrand/Berthe Rousseau

TRAINING MATERIALS

CASE SUMMARY

You are a non-English speaking person (use your own age and gender) who is complaining of extreme fatigue. Your bilingual spouse called the doctor’s office and was given a telehealth appointment. You need a translator for this appointment, as your spouse has gone to work, holding down the family restaurant.

You and your extended family own and operate Rousseau Patisserie restaurants in Las Vegas and North Las Vegas. Today is the third day in a row that you have stayed home with fatigue, fever, chills and cough. In the past 24 hours, diarrhea and runny nose have started, too. As a result, you’ve lost your appetite. You’ve had the flu before, but the severity of the fatigue is new to you. You have never felt so sick in your life.

Two weeks ago, you and your spouse returned from a dream vacation to Spain. Three days ago, the extreme fatigue set in while you were on a typical walk to the corner store. Fever, chills, and a dry cough soon followed.

You haven’t had much appetite since the runny nose started yesterday, along with the unsettled stomach and diarrhea.

Your spouse is concerned about your health. Deep down, you are too. Perhaps because it is easier on you emotionally and psychologically, you are remaining focused on keeping your business alive.

Your challenge, as the standardized patient, is:

1. To appropriately and accurately reveal the facts of the patient’s case through an interpreter in a telehealth setting.

PRESENTATION/EMOTIONAL TONE

When the student joins the video call you should be sitting in a chair wearing your regular clothes.

In general, Bertrand/Berthe, and the translator, are pleasant and easy to talk to. You answer all questions directly without ‘dancing around the subject’. Bertrand/Berthe will cough (into their elbow or a tissue) at the start of the encounter. S/He will also appear fatigued throughout the encounter.

OPENING LINE

In response to the typical opening question from the student, “What brings you in here today?” – you respond verbatim.

**“Je suis si fatigué/e et faible. Je suis à peine revenu/e de ma promenade habituelle au magasin du coin. <toux>”**

(I am so tired and weak. I barely made it back from my usual walk to the corner store. <cough>)

If invited to say more:

**“Je doutais vraiment de pouvoir passer de ma porte d'entrée à mon lit.** <si on vous le demande, **il fait environ 15 pas >**  (I really doubted I was gonna make it from my front door to my bed. < if asked, it’s 15 paces or so >)

If invited to say more:

**“J’aimerais vraiment retourner au travail.”** (I’d really like to get back to work.)

**HISTORY OF PRESENT ILLNESS (HPI)**:

*Onset:* **La fatigue a commencé il y a environ 3 jours.** Fatigue started about 3 days ago

*Duration:* **3 jours.** 3 days

*Frequency:* **Constant.** Constant

*Quality/Description:* **Je suis trop faible même pour lire le journal pendant que je suis au lit.** (I’m too weak to even read the newspaper while I’m in bed.)

*Severity/intensity:* **Je ne me suis jamais senti/e aussi malade de ma vie.** (I’ve never felt so sick in my life.)

*Location:* **Mon corps entier me fait mal.** (My whole body aches.)

*Aggravating /alleviating factors:* **Se lever pour aller aux toilettes aggrave encore les choses. Rien n'améliore la fatigue.** (Getting up to go to the bathroom makes it worse. Nothing makes the fatigue better.)

*Associated symptoms:* **Fièvre, frissons, courbatures, toux, diarrhée, un nez qui coule.** (Fever, chills, body aches, cough, diarrhea, runny nose.) If asked, the constant fever ranges from 100 to 102, the cough is constant, the diarrhea is watery (**liquide**) and happens about 4 times a day.

**RESPONSE DURING PHYSICAL EXAMINATION:** (ROM, pain, procedure responses during PE to make case clinically accurate): N/A, There is no physical examination during this case.

**Your chief concern / patient perspective of illness** (If the student asks, “What concerns you most about this?” (Or something of that nature), you reply that:

**Je veux retourner au travail le plus tôt possible.**

(I want to get back to work as soon as possible.)

If the student asks, “what effect does this have on your daily life?**” (**Or something of that nature, you reply that:

**Je ne peux pas aller travailler. L'avenir de mon entreprise et les moyens de subsistance de ma famille et de mes employés dépendent tous de moi.**  (I can’t go to work. The future of my business, and the livelihoods of my family and employees are all depending on me.)

**REVIEW OF SYSTEMS** (Items in **bold** indicate a “yes” response)

**GENERAL** – No tearing or redness of the eyes noted. **Body aches.**

**Head** – No headache

**ENT** – No tinnitus (ringing in ears), no loss of hearing. No sensitivity to noises. **Runny nose.**

**EYES:** No loss of vision, no light sensitivity**.** Past examination (in the last year) was normal.

**CV**- no chest pain, no palpitations

**LUNG** –No hemoptysis or wheezing. **Dry cough**.

**Genitourinary** – No problems with urination. No blood in the urine. Male: No erectile dysfunction. Female:(see menstrual history below**).**

**GI** – no abdominal pain. No problems with bowels, no constipation, no nausea. **Diarrhea and loss of appetite.**

**MUSCULOSKELETAL** – No joint pain or muscle pain /spasm.

**ENDOCRINE**- No hot flashes, hair loss or temperature sensitivity, no increased thirst, no recent weight loss. **Chills and** **fever**.

**SKIN**: No new rashes or other problems

**NEUROLOGIC**: No numbness, tingling, tremor, fainting, memory loss or loss of balance. **Weakness.**

**PSYCH** No flashing lights or hallucinations

**PAST MEDICAL HISTORY (PMI):**

**Past Illnesses**: **Aucun, j'ai toujours été en bonne santé.** (None, I’ve always been healthy.)

**Past surgeries**: None

**Pregnancy**: None

**Hospitalizations:** None

**Accidents/injuries: Rien de grave, entorses occasionnelles de l'enfance, etc.** (Nothing major, occasional childhood sprains, etc.)

**Immunizations: Je ne reçois pas de vaccin antigrippal annuel et je n'ai reçu aucun vaccin COVID.**

(I do not get an annual flu shot and did not get any COVID vaccinations.)

*For [female] only:*

Menstrual history: N/A

------------------------------------------------------------------------------------------------------------

**OB/GYN:** N/A

**MEDICATIONS:**

Prescriptions: **Aucun** (None)

Over-the-counter drugs: **Tylenol pour la fièvre et les courbatures.** <Si on te demande si ça aide, **je pense que ça aide un peu.>** (Tylenol for the fever and body aches. If asked whether it helps, I think it helps a little.) You’ve been taking Tylenol according to the directions on the box (2 pills every 6-8 hours) **Ce qui est indique sur la boite**.(What's indicated on the box) since the fever began. The fever peaks at 102 and the Tylenol brings it down to 100.

Herbs: **Aucun** (None)

Illicit/street drugs: **Non, jamais.** (No, never)

Allergies [Drug/other): **Aucun** (None)

**FAMILY MEDICAL HISTORY: Je viens d'une famille en très bonne santé. Nous travaillons dur et jouons dur jusqu'à ce que nous mourions de vieillesse.** (You are not aware of any major health issues in your family. “I come from a very healthy family. We work hard and play hard until we die of old age.)

As the ages of the SPs portraying this case will vary, so will the ages and health status of their relatives. Please take time to fill in this portion with the ages and health status (either “Alive and Healthy” or “Deceased of Old Age”) of your imaginary family in keeping with your real age.

Father:

*age*

*Health status/history*

Mother:

*age*

*Health status/history*

Sibling(s):

*age*

*Health status/history*

Grandfather (paternal):

*age*

*Health status/history*

Grandmother (paternal):

*age*

*Health status/history*

Grandfather (maternal):

*age*

*Health status/history*

Grandmother (maternal)

*age*

*Health status/history*

**PRESENT LIVING SITUATION**

**J'ai une maison ici à Las Vegas où je vis avec mon conjoint/e. Nous avons deux enfants adultes.**

(You live in a house in Las Vegas with your spouse. Your 2 children are grown.) or (**Nous avons deux jeunes enfants qui font l'ecole.** - We have two young children who go to school.)

If asked about sick contacts, i.e. Have you been around anyone who is sick? At home? At work?, you reply:

**Pas que je sache, mais c’est possible. Quand je vois quelqu'un qui a le nez qui coule, qui éternue, tousse, je pense généralement que c’est la saison des allergies. Maintenant je ne suis pas si sûr/e…**

(Not that I’m aware of, but it’s possible. When I see someone with a runny nose, sneezing, coughing, I usually just figure it’s allergy season. Now I’m not so sure…)

**SOCIAL HISTORY:**

*Occupation:* **Je possède un restaurant. J'espère que nous ne ferons pas faillite.**  (I own a restaurant. I hope we don’t go out of business.)

*Marital Status:* **Marié/e** (Married)

*Support system:* **J'ai un excellent système de soutien de la famille soudée et des amis proches qui sont comme la famille.** (Large support system of close-knit family and friends who are like family.)

*Sleep pattern:* **Je n’avais aucun mal à dormir ces derniers temps. J'ai toujours bien dormi, 6 à 8 heures par nuit.** (I haven’t had any trouble sleeping lately*.* I’ve always slept well, 6 -8 hours each night)

*Alcohol :* **3-4 verres par semaine** (3-4 drinks a week)

*Tobacco :* **Non, jamais.** (No, never)

*Diet :* **Je mange une alimentation équilibrée au restaurant et à la maison. Il n'y a que de grands cuisiniers dans ma famille.** (I eat a balanced diet at the restaurant and at home. There are nothing but great cooks in my family.)

*Caffeine :* **1[une] tasse chaque matin avec petit-déjeuner .** (1 cup each morning with breakfast)

*Exercise*: **Je prends soin de moi, je me promène et je suis debout toute la journée au travail.** (I take care of myself, I take walks, and I’m on my feet all day at work.)

*Activities/hobbies :* **Je passe du temps avec ma famille. Jouer avec mes petits-enfants. Allez aux matchs de football de ma famille.** Spending time with my family. Playing with my grandkids. Going to my family’s soccer games.

*Travel :* **Mon mari/ma femme et moi sommes revenus d'Espagne il y a 2 semaines.** (My husband/wife and I returned from Spain 2 weeks ago.)

*Sexual History:* **Je suis actif/ve avec mon conjoint/e.** (I’m active with my spouse.)

*Spirituality / Religion:* **J'ai été élevé/e catholique et j'ai élevé mes enfants catholiques.** (I was raised Catholic and raised my kids Catholic.)

The two questions an SP can ask for this case are:

1. (Dx related) **Quel est le problème selon vous?** (What do you think the problem is?)
2. (Educational) **Quand puis-je retourner au travail?** (When can I go back to work?)

***Interpreter Information***

Beto/Berta Ruiz

TRAINING MATERIALS

**Reason for your today’s visit*-(Quelle est la raison de ta visite aujourd'hui?)***

**When did you start having symptoms*-(Quand as-tu commencé à avoir des symptômes ?)***

**How many days have you felt like this*-(Combien de jours as-tu ressenti comme ça?)***

**What is the frequency of your symptoms*-(Quelle est la fréquence de tes symptômes?)***

**Please describe your symptoms*-(*** ***S'il te plaît décris tes symptômes ?)***

**Do you have a cough*-(As-tu une toux?)***

**How frequently do you cough?*(À quelle fréquence tousses-toi?)***

**Is it productive or dry*- (Est-ce productif ou sec?)***

**Do you have a fever*-(As-tu de la fièvre ?)***

**How high is your fever*- (Quelle est ta fièvre?)***

**What is the pattern of your fever?** ***(Quel est le schéma de ta fièvre?)***

**Did the Tylenol reduce your fever?** ***(Le Tylenol a-t-il réduit ta fièvre?)***

**How much Tylenol are you taking?** ***(Combien de Tylenol prends-tu?)***

**Do you have shortness of breath*- (As-tu un essoufflement?)***

**Can you breathe- *(Peux-tu respirer?)***

**What is the severity or intensity of your pain? *- (Quelle est la gravité ou l'intensité de ta douleur?)***

**What are your aggravating or alleviating factors - *(Quels sont tes facteurs aggravants ou atténuants?)***

**Associated symptoms*- (Quels sont tes symptômes associés?)***

**How frequently are you having diarrhea?** ***(À quelle fréquence as-tu la diarrhée?)***

**Describe the diarrhea** ***(Décris la diarrhée)***

**What is the consistency of it?** ***(Quelle est la consistance de celui-ci?)***

**Previous episodes*-(Avoir eu ces symptômes dans le passé?)***

**What worries you the most about this illness*-(Qu'est-ce qui t’inquiète le plus de cette maladie?)***

**What effect does this have on your daily life? *- (Quel effet cela a-t-il sur ta vie quotidienne?)***

**Past illnesses*- (As-tu eu des maladies dans le passé?)***

**Past surgeries- *(As-tu subi des chirurgies dans le passé?)***

**Pregnancies? *- (Grossesses?)***

**Hospitalizations*-(As-tu eu des hospitalisations?)***

**Accidents/injuries*-(As-tu eu des accidents / blessures?)***

**Immunizations*-( Tes vaccinations sont-elles à jour?)***

**Do you take medication-*(Prends-toi des médicaments?)***

**Over-the-counter drugs*-(Prends-toi des médicaments en vente libre?)***

**Herbs*-(Prends-tu des herbes?)***

**Illicit/street drugs*-(Utilises-toi des drogues illicites?)***

**Do you have allergies*-(As-tu des allergies?)***

**Are you allergic to any medications*-(Es-tu allergique à des médicaments?)***

**What is your family medical history*-(Quels sont tes antécédents médicaux familiaux?)***

**In the past two weeks have you had contact with anyone who is sick? At home? At work? *- (Au cours des deux dernières semaines, as-tu eu des contacts avec une personne malade? À la maison? Au travail?)***

**Occupation*-(Quel est ton occupation?)***

**Support system- *(Quel est ton système de soutien?)***

**Sleep pattern*-(Comment dors-tu bien la nuit?)***

**Alcohol*- (Est ce que tu bois de l'alcool? À quelle fréquence?)***

**Tobacco*-(Fumes-toi des cigarettes ou utilises-toi du tabac?)***

**Diet- *(Comment est ton alimentation?)***

**Caffeine:**-***(Quelle est ta consommation quotidienne de caféine?)***

**Exercise-*( Fais-tu de l'exercice?)***

**Activities/hobbies-*(Quelles sont tes activités et tes passe-temps en dehors du travail?)***

**Sexual History*-(Quelle est ton histoire sexuelle? Es-tu sexuellement actif/ve ??)***

**Spirituality / Religion*-(Quelle est ta religion?)***
