# Supplementary material for: Social Determinants of Health: A Multilingual Standardized Patient Case to Practice Interpreter Use in a Telehealth Visit
Source: MedEdPORTAL. 2023 Nov 14;19:11364. doi: 10.15766/mep_2374-8265.11364 (PMC10643468; doi:10.15766/mep_2374-8265.11364)
Supplement: Supplementary file 1 — SP Case - Spanish.docxSP Case - Tagalog.docxSP Case - Igbo.docxSP Case - French.docxSMI - Spanish.docxSMI - Tagalog.docxSMI - Igbo.docxSMI - French.docxSPL Rehearsal Script.docxDoor Instructions - Spanish and Tagalog.docxDoor Instructions - Igbo.docxDoor Instructions - French.docxFaculty Guide.pdfStudent Guide.pdfImportant Points Interpreters Telehealth.docxGraphic Instructional Tool.pdfSample Progress Note.docxProgress Note Grading Rubric.xlsx [file mep_2374-8265.11364-s001.zip › I. SPL Rehearsal Script.docx]

**MEDICAL INTERVIEW TOPICS**

INTRODUCTION

- Verify patient identity
- Secure interpreter
- Introduce self

CHIEF CONCERN (Why are you being seen today?)

- Follow-up as appropriate

HISTORY OF PRESENT ILLNESS

- Onset
- Duration
- Frequency
- Quality/description
- Severity/intensity
- Location
- Aggravating/alleviating factors
- Associated symptoms

REVIEW OF SYSTEMS

- General
- Head
- Eyes
- ENT
- CV (heart)
- Lung
- GI (gastro-intestinal)
- GU (genitourinary)
- MSK (musculoskeletal)
- Endocrine
- Skin
- Neurologic
- Psych

PAST MEDICAL HISTORY

- Past illnesses (diagnoses, chronic issues)
- Past surgeries
- Pregnancies
- Hospitalizations
- Accidents/injuries
- Immunizations

MEDICATIONS

- Prescriptions
- Over the counter
- Supplements/herbs
- Illicit/recreational drugs
- Allergies (drugs & other)

FAMILY MEDICAL HISTORY

- Father
- Mother
- Siblings
- Grandparents

PRESENT LIVING SITUATION

- Location (urban, suburban, remote)
- Type of building (house, apartment, etc.)
- Others in the dwelling?
- Recent environmental changes

SOCIAL HISTORY

- Occupation
- Education level
- Marital/relationship status
- Support system
- Sleep pattern/habits
- Alcohol
- Tobacco
- Diet
- Caffeine
- Exercise
- Activities/hobbies
- Travel
- Sexual history
- Spirituality/religion

PATIENT PERSPECTIVE (example queries)

- What concerns you most about this?
- How does this affect your daily life?
- What might you think is going on?

PHYSICAL EXAM – n/a

**SAMPLE SCRIPT A**

- Introduction
- What brings you in today?
- How many days have you felt like this?
- Will you please describe your symptoms
- Do you have a cough?
  - Is it productive? Or dry
- Do you have a fever?
  - How high is your fever?
  - What is the pattern of your fever?
  - Did the Tylenol reduce your fever?
  - How much Tylenol are you taking?
- Do you have shortness of breath?
  - Can you breathe?
- What is the intensity of your pain?
- Does anything make it better?
- Does anything make it worse?
- Do you have any other symptoms?
  - How frequently are you having diarrhea?
  - Describe the diarrhea.
  - Is there any blood in it?
  - What is the consistency of it?
- Have you had anything like this in the past?
- Tell me about your past illnesses.
- Have you had any surgeries?
- Pregnancies?
- Prior hospitalizations
- Accidents or injuries
- Do you take any medications?
- Over the counter drugs
- Herbs
- Illicit or street drugs
- Tobacco use
- Alcohol consumption
- Do you have any allergies?
- Tell me about your diet
- Tell me about your caffeine intake
- Do you exercise?
- In the past two weeks have you had any contact with someone who is sick?
  - At home?
  - At work/
- What questions do you have for me?

**SAMPLE SCRIPT B**

- Introduction
- What brings you in today?
- How many days have you felt like this?
- Will you please describe your symptoms
- Do you have a cough?
  - Is it productive? Or dry
- Do you have a fever?
  - How high?
  - For how long?
- Do you have shortness of breath?
- Does anything make it better or worse?
- What other symptoms do you have?
  - How often are you having diarrhea?
  - Can you describe the diarrhea?
- Have you had anything like this in the past?
- Tell me about your past illnesses.
- Have you had any surgeries?
- Pregnancies?
- Prior hospitalizations
- Accidents or injuries
- Do you take any medications?
- Over the counter drugs
- Do you drink or smoke?
- Illicit or street drugs
- Do you have any allergies?
- What do you do for a living?
- Are you married?
- Are your parents still living? How is their health?
- Tell me about your diet
- Have you been sleeping regularly?
- In the past two weeks have you had any contact with someone who is sick?
  - At home?
  - At work?
- What concerns you most about this?
- Do you have any questions for me?
