# Supplementary material for: Social Determinants of Health: A Multilingual Standardized Patient Case to Practice Interpreter Use in a Telehealth Visit
Source: MedEdPORTAL. 2023 Nov 14;19:11364. doi: 10.15766/mep_2374-8265.11364 (PMC10643468; doi:10.15766/mep_2374-8265.11364)
Supplement: Supplementary file 1 — SP Case - Spanish.docxSP Case - Tagalog.docxSP Case - Igbo.docxSP Case - French.docxSMI - Spanish.docxSMI - Tagalog.docxSMI - Igbo.docxSMI - French.docxSPL Rehearsal Script.docxDoor Instructions - Spanish and Tagalog.docxDoor Instructions - Igbo.docxDoor Instructions - French.docxFaculty Guide.pdfStudent Guide.pdfImportant Points Interpreters Telehealth.docxGraphic Instructional Tool.pdfSample Progress Note.docxProgress Note Grading Rubric.xlsx [file mep_2374-8265.11364-s001.zip › J. Door Instructions - Spanish and Tagalog.docx]

PRESENTING SITUATION

and

INSTRUCTIONS TO THE STUDENT

Beto/Berta Ruiz

Beto/Berta Ruiz is an adult male or female who has been told to call TELEHEALTH SERVICES today for extreme fatigue. The patient does not speak English.

Vital signs:

T: 102° F oral Pulse: 75 bpm BP: 132/64 RR: 25

You are to:

- Develop ways to create an environment conducive to conducting a telehealth visit that includes an interpreter.
- Demonstrate appropriate history gathering and physical exam components while interviewing a patient with fatigue during a telehealth visit.
- Apply techniques from the interpreter services reference materials to interview a non-English language preference patient with an interpreter and critique a peer after observing.
- Integrate information from the case and faculty and peer feedback to create a progress note with an appropriate basic differential diagnosis and treatment plan for a patient with fatigue.
