# Supplementary material for: Social Determinants of Health: A Multilingual Standardized Patient Case to Practice Interpreter Use in a Telehealth Visit
Source: MedEdPORTAL. 2023 Nov 14;19:11364. doi: 10.15766/mep_2374-8265.11364 (PMC10643468; doi:10.15766/mep_2374-8265.11364)
Supplement: Supplementary file 1 — SP Case - Spanish.docxSP Case - Tagalog.docxSP Case - Igbo.docxSP Case - French.docxSMI - Spanish.docxSMI - Tagalog.docxSMI - Igbo.docxSMI - French.docxSPL Rehearsal Script.docxDoor Instructions - Spanish and Tagalog.docxDoor Instructions - Igbo.docxDoor Instructions - French.docxFaculty Guide.pdfStudent Guide.pdfImportant Points Interpreters Telehealth.docxGraphic Instructional Tool.pdfSample Progress Note.docxProgress Note Grading Rubric.xlsx [file mep_2374-8265.11364-s001.zip › K. Door Instructions - Igbo.docx]

PRESENTING SITUATION

and

INSTRUCTIONS TO THE STUDENT

Ogo Azunna

Ogo Azunna is an adult male or female who has been told to call TELEHEALTH SERVICES today for extreme fatigue. The patient does not speak English.

Vital signs:

T: 102° F oral Pulse: 75 bpm BP: 132/64 RR: 25

You are to:

- Develop ways to create an environment conducive to conducting a telehealth visit that includes an interpreter.
- Demonstrate appropriate history gathering and physical exam components while interviewing a patient with fatigue during a telehealth visit.
- Apply techniques from the interpreter services reference materials to interview a non-English language preference patient with an interpreter and critique a peer after observing.
- Integrate information from the case and faculty and peer feedback to create a progress note with an appropriate basic differential diagnosis and treatment plan for a patient with fatigue.
