# Supplementary material for: Social Determinants of Health: A Multilingual Standardized Patient Case to Practice Interpreter Use in a Telehealth Visit
Source: MedEdPORTAL. 2023 Nov 14;19:11364. doi: 10.15766/mep_2374-8265.11364 (PMC10643468; doi:10.15766/mep_2374-8265.11364)
Supplement: Supplementary file 1 — SP Case - Spanish.docxSP Case - Tagalog.docxSP Case - Igbo.docxSP Case - French.docxSMI - Spanish.docxSMI - Tagalog.docxSMI - Igbo.docxSMI - French.docxSPL Rehearsal Script.docxDoor Instructions - Spanish and Tagalog.docxDoor Instructions - Igbo.docxDoor Instructions - French.docxFaculty Guide.pdfStudent Guide.pdfImportant Points Interpreters Telehealth.docxGraphic Instructional Tool.pdfSample Progress Note.docxProgress Note Grading Rubric.xlsx [file mep_2374-8265.11364-s001.zip › M. Faculty Guide.pdf]

UNLV | School of MEDICINE

Doctoring Faculty Guide

Session ***

Doctoring Session ***

Semester Year

Location Date of session

Online only

Telehealth and Interpreter services

Learning Objectives

By the end of the session, students will be able to:

1. Develop ways to create an environment conducive to conducting a telehealth visit that includes an interpreter.
2. Demonstrate appropriate history gathering and physical exam components while interviewing a patient with fatigue during a telehealth visit.
3. Apply techniques from the interpreter services reference materials to interview a non-English language preference patient with an interpreter and critique a peer after observing.
4. Integrate information from the case and faculty and peer feedback to create a progress note with an appropriate basic differential diagnosis and treatment plan for a patient with fatigue.

Activities

1. Remote view and perform in pairs a case via Zoom with your group during class time.
2. Comment during the case with chat and debrief after each demonstration of the case regarding use of interpreters, an initial approach to a patient with fatigue, virtual factors that affected the interview, and what physical exam elements could be accomplished.
3. After class, write up the case in SOAP note format. Submit via Canvas by the due date.

Materials:

- Computer access

Pre-session assignments:

1. Review Lange: chapter 7: Fatigue
2. Review “Important Points About Interpreters and Telehealth” in Canvas.
3. Review “The Telehealth Ten: A Guide for Patient-Assisted Virtual Physical Examination” in Canvas.
4. Optional: Review the Graphic Instructional Tool in Canvas

Post-Session Assignments:

1. **Write up the case in SOAP format** using the link for progress notes. Submit to Canvas by [date].

Evaluation:

Students will submit a progress note after class and receive written faculty feedback and a rubric score.

Faculty Instructions:

Dear Faculty,
The case is a Spanish, Tagalog, Igbo, or French speaking patient with fatigue and URI symptoms (see case in Canvas) that is having a telehealth visit.
This will be our first attempt interviewing a live SP via Zoom. You will meet with your two groups separately during your assigned times (8 am and 10 am).

In preparation for the session:

1. Review the diagnostic approach to fatigue in Lange Ch. 7. In particular there is an algorithm for a patient with fatigue and a differential.
2. Please review the Canvas materials regarding interpreters and telehealth tips listed in Pre-session Assignments section above.

The session:

Starting the meeting:

1. Have important contacts by you in case of technical issues:
   Name, phone number   Name, phone number

2. Have your two group meeting links available (located in Canvas) Each Group has a separate Zoom meeting link and separate pairs of standardized patients (the patient and the interpreter). Groups 1-5 start at 8 am, groups 6-10 at 10 am.

3. The session Zoom meetings were initially set for two hours. That said, some free accounts may log out after 40 min. Let your students know to just log back in if that happens. Log out and back in the other group link for the second session.

4. Make sure the links are set up with you as faculty already as the host. When you arrive in the meeting if someone else created the meeting, they have to be there and should transfer the host role to you. This is problematic if the same person has to go room to room transferring all the hosts. For transferring host: host should open "participants" at the bottom and then hover over the name of the person to be made host and select from the drop down.

5. The students will remain in the waiting room while the faculty quickly orients the SPs. The faculty can open Chat and send a message to the waiting room to wait while they orient the SPs.

Orienting the SPs

6. You will bring from the Zoom waiting room the two standardized patients and take less than two minutes to
    - Introduce yourself to the SPs and make sure you know which SP is playing the patient and which is the interpreter so you invite the patient in first and the interpreter later when the student asks for the interpreter. You may need to change SP's name label on Zoom to the case name (e.g., Beto or Berta Ruiz) and Interpreter. You can do this as the host by hovering over the participant and dropping down to rename.
    - Make sure the lighting, positioning of the SPs, and audio are optimal.
    - Let them know you need 10 minutes to orient the students and select the order of interviewers.
    - Let the SPs know the next time they will enter with the interviewers in place.The patient first and then the interpreter when the student requests one.
    - Then put both SPs back in the virtual waiting room hovering and selecting "put back in waiting room"

Orienting the student learners

7. Faculty will bring the group of students from the waiting room into the virtual room.
8. Faculty will select two students for the initial interview. One student can do the chief complaint, HPI, ROS, and past medical history. The second student can complete the social history (may need to be limited depending on time) and whatever inspection exam that is possible and then discuss an assessment and plan.The time estimate is 20 minutes and faculty should remind students it takes twice as long with the interpreter.
9. The faculty should make sure the selected students do not speak the language that will be interpreted.
10. Faculty will review the door note with learning objectives again with the students, so faculty should have it available.
11. The faculty will explain that the first student will need to request an interpreter during the case and request the language of the interpreter. Students will then need to orient the interpreter to the patient. Student should ask the interpreter to introduce themselves again.
12. The students should attempt explaining an assessment and plan to the patient.

During the encounter:

13. During the encounter faculty and the observing students are encouraged to chat when elements of either proper or improper interpreter use are observed as well as elements related to fatigue or the telehealth environment. Remember most of the critiquing can happen in the chat rather than waiting for the debrief time afterwards. The student currently interviewing will not be expected to look at the chat.

Beginning the case

14. The faculty will chat to the SPs in the waiting room that they are beginning the session. The faculty will then admit the "patient" out of the waiting room into the main room and the interview begins. All students except the interviewer should mute their microphone.

15. The student should proceed with the interview. Once the student notices that they need an interpreter, they can request one. They can simply tell the patient they are going to get an interpreter. The faculty will then allow the interpreter in the room. The interview will continue. The second student keeps the camera off until the first student references them "my colleague will now be joining us". First student remains in the room as well. All other students should have cameras off and be muted. (Tip: If the first student is taking too long, the second student can just turn on the camera and wait to be introduced). When it is their turn, the second student will quickly introduce themselves and continue (don't introduce both students at the beginning).

Ending the case:

16. Once the interview is finished, the faculty will remove the patient (hover over participant and select remove) and interpreter from the meeting room, and at this point the debrief with the students will begin. DO NOT SELECT END MEETING. Ideally, the faculty does not interject or give feedback until the SPs are released from the room.

The Debrief:

17. Things to discuss: How did the interviewers feel about the visit: What went well? What could be improved? Were there any missing elements from the history, physical, assessment and plan? Give the other students an opportunity to give some feedback.

Ending the meeting:

18. Prior to ending the meeting, faculty will remind students they need to complete a note in Canvas on the case which will be graded with the rubric.

<u>Example Schedule</u>

Group 1 5-6 students 8 am - 10 am

8:00 Brief instructions to the SP - checking labels, sound, lighting.
8:02 Orient the students, select interviewers, encourage chat observations.
8:15 Start case Round 1
8:35 Debrief with faculty (10 min first debrief)
8:45 Start case Round 2

9:05 Debrief with faculty (5 min)
9:10 Start case Round 3
9:30 Debrief with faculty (5-20 min)
End of session (9:50)

Group 2 5-6 students 10 am - 12 pm

10:00 Brief instructions to the SP - checking labels, sound, lighting.
10:02 Orient the students, select interviewers, encourage chat observations.
10:15 Start case Round 1
10:35 Debrief with faculty (10 min first debrief)
10:45 Start case Round 2
11:05 Debrief with faculty (5 min)
11:10 Start case Round 3
11:30 Debrief with faculty (5-20 min)
End of session (11:50)
