# Supplementary material for: Social Determinants of Health: A Multilingual Standardized Patient Case to Practice Interpreter Use in a Telehealth Visit
Source: MedEdPORTAL. 2023 Nov 14;19:11364. doi: 10.15766/mep_2374-8265.11364 (PMC10643468; doi:10.15766/mep_2374-8265.11364)
Supplement: Supplementary file 1 — SP Case - Spanish.docxSP Case - Tagalog.docxSP Case - Igbo.docxSP Case - French.docxSMI - Spanish.docxSMI - Tagalog.docxSMI - Igbo.docxSMI - French.docxSPL Rehearsal Script.docxDoor Instructions - Spanish and Tagalog.docxDoor Instructions - Igbo.docxDoor Instructions - French.docxFaculty Guide.pdfStudent Guide.pdfImportant Points Interpreters Telehealth.docxGraphic Instructional Tool.pdfSample Progress Note.docxProgress Note Grading Rubric.xlsx [file mep_2374-8265.11364-s001.zip › N. Student Guide.pdf]

Doctoring Student Guide
Session ***

Doctoring Session *
Semester Year
Location Date of session
Online only

Telehealth and Interpreter services

Learning Objectives

By the end of the session, students will be able to:
1. Develop ways to create an environment conducive to conducting a telehealth visit that includes an interpreter.
2. Demonstrate appropriate history gathering and physical exam components while interviewing a patient with fatigue during a telehealth visit.
3. Apply techniques from the interpreter services reference materials to interview a non-English language preference patient with an interpreter and critique a peer after observing.
4. Integrate information from the case and faculty and peer feedback to create a progress note with an appropriate basic differential diagnosis and treatment plan for a patient with fatigue.

Activities

1. Remote view and perform in pairs a case via Zoom with your group during class time.
2. Comment during the case with chat and debrief after each demonstration of the case regarding use of interpreters, an initial approach to a patient with fatigue, virtual factors that affected the interview, and what physical exam elements could be accomplished.
3. After class, write up the case in SOAP note format. Submit via Canvas by the due date.

Evaluation:

Students will submit a progress note after class and receive written faculty feedback and a rubric score.

Materials:

- Computer access

Pre-session assignments:

1. Review Lange: chapter 7: Fatigue
2. Review “Important Points About Interpreters and Telehealth” in Canvas.
3. Review “The Telehealth Ten: A Guide for Patient-Assisted Virtual Physical Examination” in Canvas.
4. Optional: Review the Graphic Instructional Tool in Canvas

Post-Session Assignments:

Write up the case in SOAP format using the link for progress notes. Submit to Canvas by [date].
