# Supplementary material for: Social Determinants of Health: A Multilingual Standardized Patient Case to Practice Interpreter Use in a Telehealth Visit
Source: MedEdPORTAL. 2023 Nov 14;19:11364. doi: 10.15766/mep_2374-8265.11364 (PMC10643468; doi:10.15766/mep_2374-8265.11364)
Supplement: Supplementary file 1 — SP Case - Spanish.docxSP Case - Tagalog.docxSP Case - Igbo.docxSP Case - French.docxSMI - Spanish.docxSMI - Tagalog.docxSMI - Igbo.docxSMI - French.docxSPL Rehearsal Script.docxDoor Instructions - Spanish and Tagalog.docxDoor Instructions - Igbo.docxDoor Instructions - French.docxFaculty Guide.pdfStudent Guide.pdfImportant Points Interpreters Telehealth.docxGraphic Instructional Tool.pdfSample Progress Note.docxProgress Note Grading Rubric.xlsx [file mep_2374-8265.11364-s001.zip › O. Important Points Interpreters Telehealth.docx]

**Important Points Regarding Interpreters and Telehealth**

**Interpreter Tips:**

**Why is it important to have a trained interpreter?**

- In a study reviewing visits conducted with non-English language preference (NELP) patients without an interpreter, an average of 31 errors in communication occurred per visit.
- Studies show patients from racial and ethnic minority groups, especially NELP, are less likely to engender empathic responses from physicians, establish rapport with physicians, receive sufficient information, and be encouraged to participate in medical decision-making.

**How do you choose a quality interpreter?**

- Not a family member
- NEVER a child
- Fluent in both languages including medical terminology
- Professional training in interpretation
- Confidential, impartial, complete, accurate
- Act as a cultural broker and patient advocate

**What are some of the consequences if a trained interpreter is not used?**

- Errors: wrong diagnosis; wrong treatment
- Adverse outcome for patient
- Ordering unneeded diagnostic tests
- Lack of patient understanding, compliance, and satisfaction
- Lack physician satisfaction
- Lawsuit

**How do you communicate effectively through an interpreter?**

- Introduce yourself and orient the interpreter
- Position the interpreter behind you OR beside the patient, NOT between you if in person. If using telehealth, make sure all participants are on gallery view.
- Speak directly to the patient
- Speak at an even pace in short segments; pause so the interpreter can interpret
- Avoid asking multiple questions or complicated compound sentences
- Avoid phrases particular to English
- Ask the patient to repeat back their understanding of what has been said (discharge/medication instructions)
- Try to gather the history as you would for any other patient. Ask questions and allow the patient to talk.
- Be patient; Don’t give up.
- Encourage the interpreter to alert you to cultural concepts or misunderstandings

**Telehealth Tips:**

**Patient preparation:**

- Ensure the patient receives directions on how to access the virtual waiting room and suggest a “dry run”.
- Include directions to the patient if the patient is expected to obtain vital signs prior to the appointment.

**Visit initiation:**

- Confirm audio and video function.
- Address any distractions, issues with the environment.
- Introduce yourself and confirm patient identity with two identifiers.
- Inquire if the patient has a caregiver or other family members to assist if needed and ensure appropriate privacy/confidentiality.
- Ask if interpreter services are needed.

**Physical Assessment:**

- Ensure appropriate privacy to perform exams.
- Direct the patient to position themselves to facilitate visual inspection.
- When directing patients to examine for lumps or tenderness, ask patients to describe what they are feeling.
- Ensure safety if asking patients to stand or walk.
- Document which parts of the exam were observed and which were patient reported.

Reference:

This instructional tool was created by Laura Shaw MD in April of 2020 and updated in 2023 by adapting information from the following presentation : Effective Communication through an Interpreter and Introduction to Cultural Competence ©Yvonne T. Murphy, M.D. 2011 and article: Benziger CP, Huffman MD, Sweis RN, Stone NJ. The telehealth ten: a guide for a patient- assisted virtual physical examination. Am J Med.2021 Jan;134(1)48-51. Doi: 1016/j.amjmed.2020.06.015
