# Supplementary material for: Social Determinants of Health: A Multilingual Standardized Patient Case to Practice Interpreter Use in a Telehealth Visit
Source: MedEdPORTAL. 2023 Nov 14;19:11364. doi: 10.15766/mep_2374-8265.11364 (PMC10643468; doi:10.15766/mep_2374-8265.11364)
Supplement: Supplementary file 1 — SP Case - Spanish.docxSP Case - Tagalog.docxSP Case - Igbo.docxSP Case - French.docxSMI - Spanish.docxSMI - Tagalog.docxSMI - Igbo.docxSMI - French.docxSPL Rehearsal Script.docxDoor Instructions - Spanish and Tagalog.docxDoor Instructions - Igbo.docxDoor Instructions - French.docxFaculty Guide.pdfStudent Guide.pdfImportant Points Interpreters Telehealth.docxGraphic Instructional Tool.pdfSample Progress Note.docxProgress Note Grading Rubric.xlsx [file mep_2374-8265.11364-s001.zip › Q. Sample Progress Note.docx]

Sample Student Progress Note:

**Question 1**

**/ 24 pts**

**Name of Case:  B. Ruiz**

**History** - Describe the history you just obtained from this patient.  Include only information relevant to this patient's problem(s).  Include pertinent positives and negatives.  Start with ID, CC, HPI, Major and pertinent minor criteria.

Your Answer:

ID: Berta Ruiz. 40-year-old female

CC: “Tired and weak”

HPI: Berta Ruiz is a Spanish-only speaker and all communication was done through an official interpreter. Mrs. Ruiz has a telehealth visit with our clinic for fatigue and weakness that began 3 days ago. The onset of fatigue and weakness was sudden. Patient first noticed these symptoms as she had difficulty completing her walk home from a nearby corner store. The patient also describes associated body aches and fever. Any activity, including using the restroom, exacerbates her fatigue. Patient tried Tylenol, which helped reduce her fever, but did not alleviate her fatigue. The patient explains that her weakness is affecting both her arms and legs. When asked to describe and rate the pain of the body aches, the patient states that her “whole body hurts” and she has “never felt so sick in her life.” Patient’s primary concern is that she is unable to go to work and her family is depending on her.

ROS:

- Constitutional: Fatigue, fever, body aches, chills, loss of appetite
- GI: Diarrhea. Negative for nausea, vomiting, and abdominal pain.
- Neurological: **Loss of taste and smell (began 1 week ago)***. Weakness. Negative for headaches and dizziness.
- HENT: Rhinorrhea (began 1 day ago)
- Respiratory: Dry cough (began 1 day ago). Negative for chest pain and shortness of breath.
- Eyes: No vision changes.

Past Medical History:

- No chronic disorders.
- Minor illnesses in the past, but recovered completely.
- Vaccination record is up to date except for COVID vaccine.

Past Surgical History: None

Hospitalizations: Twice during vaginal childbirth.

Family History: None. Patient states that all family members who have passed away have done so from old age.

Social and Personal history:

- Returned from a 5-day-trip to New York City two weeks ago. Trip was cut short from 10 days due to fear of new Coronavirus variants.
- When asked about sick contacts, the patient states that she had been around people who were sniffling, but she assumed it was seasonal allergies.
- Works as the owner of a restaurant. Normally works every day, but has not worked for the past 3 days due to symptoms.
- Patient cooks along with other members of her family. Diet consists of vegetables, soups, beans, and cheese.
- Drinks 3 to 4 alcoholic beverages per week.
- Married to her husband for 15 years with whom she is monogamous.
- Patient has two children aged 8 and 12. Both attend school online.
- Exercise: Patient walks frequently and is on her feet all day at the restaurant.
- Never smoked
- Never used recreational drugs.
- Spirituality: Patient is Catholic

Current medications:

- No prescriptions
- Over the counter: 2 Tylenol pills every six hours.

Allergies:

- No known drug allergies
- No seasonal or food allergies

Additional Comments:

**Question 2**

**/ 3 pts**

**Physical Exam Findings** -Describe any positive and negative findings relevant to this patient's problem(s).  Be careful to include ONLY those parts of examination you witnessed watching in THIS encounter.  Please note the vitals from the document provided before the case entitled "presenting situation and instructions to the learner."

Your Answer:

Vital signs:

Temperature: 102 degree F taken orally

BP: 132/64 mmHg

Pulse: 75bpm

RR: 25/min

General: Tired-appearing woman who is coughing throughout the visit.

Virtual pulmonary exam: Patient is able to speak in complete sentences without needing to pause for breath. No visible use of accessory respiratory muscles.

Additional Comments:

**Question 3**

**/ 6 pts**

Based on what you have learned from the history and physical examination, list diagnoses that might explain this patient's complaint(s).  List your diagnoses from most to least likely. Then, enter the positive of negative findings from the history and the physical examination (if present) that support each diagnosis.  Please list as below:

**Data Interpretation (Assessment)**

**First diagnosis**

History elements supporting primary diagnosis

Physical exam elements supporting primary diagnosis

**Second diagnosis**

History elements supporting secondary diagnosis

Physical exam elements supporting secondary diagnosis

**Third diagnosis**

History elements supporting tertiary diagnosis

Physical exam elements supporting tertiary diagnosis

Answer:

The most likely diagnosis is COVID-19*

History supporting COVID-19 diagnosis: The patient’s history of recent travel, exposure to many different people every day (restaurant), and potential recent sick contacts make an infectious disease the most likely diagnosis. The patient’s symptoms of fatigue, fever, body aches, chills, loss of appetite, weakness, diarrhea, rhinorrhea, and cough all support an infectious cause of the patient’s symptoms. The patient’s losses of her sense of taste and smell is specific to COVID-19. Furthermore, the timing of the patient’s visit during the Coronavirus pandemic and the fact that the patient has not received the COVID-19 vaccine also supports this diagnosis.

Physical exam findings supporting COVID-19: Tired-appearing woman who is coughing throughout the visit.

A second possible diagnosis is Influenza

History supporting Influenza diagnosis: The patient’s history of recent travel, exposure to many different people every day (restaurant), and potential recent sick contacts make an infectious disease the most likely diagnosis. The patient’s symptoms of fatigue, fever, body aches, chills, loss of appetite, weakness, diarrhea, rhinorrhea, and cough all support an infectious cause of the patient’s symptoms.

Physical exam findings supporting Influenza diagnosis: Tired-appearing woman who is coughing throughout the visit.

A third possible diagnosis is Pneumonia

History supporting Pneumonia diagnosis: The patient’s history of recent travel, exposure to many different people every day (restaurant), and potential recent sick contacts make an infectious disease the most likely diagnosis. The patient’s symptoms of fatigue, fever, body aches, chills, loss of appetite, weakness, diarrhea, rhinorrhea, and cough all support an infectious cause of the patient’s symptoms. Lack of productive cough and chest pain make COVID-19 and influenza more likely than pneumonia or acute bronchitis.

Physical exam findings supporting Pneumonia diagnosis: Tired-appearing woman who is coughing throughout the visit.

Additional Comments:

**Question 4**

**/ 4 pts**

**PLAN**

**Labs/Diagnostic Studies:**List initial diagnostic studies/data collection (if any) you would order/suggest for this patient. *Each study/recommendation should be appropriately linked to a diagnosis from question 3.*

**Medications/Treatments:** prescribe any medications/durable medical goods (splints/crutches, etc), physical therapy, counseling. **Any specific prescription medications should be written as a prescription.**

**Patient Education:**regarding lifestyle modification (if appropriate)

**Follow up Plan:**  **SPECIFIC** follow-up interval **and reason for follow-up**/disposition.

Answer:

Labs/Imaging/Diagnostic Studies:

- COVID-19 test for active infection
- Influenza virus test for active infection

Medications/Treatments: None at this time

Patient Education: Patient needs to isolate for 3 days from the time of visit and until she is afebrile for a period of 24 hours. Patient must rest in a room in her house/apartment during this time. She and other household members must wear a mask when in close proximity to one another and all members of the household must wash their hands frequently and diligently. If the patient experiences worsening shortness of breath, she must go to the emergency room to seek urgent medical care.

Follow up Plan: Follow up in one week or sooner if patient experiences worsening shortness of breath. Follow up visit should evaluate changes in symptoms, but additional COVID/influenza testing should not be done. Follow up visit should also include a discussion about any new symptoms that the patient’s family members may be experiencing, and considering isolation for them if they develop symptoms.

Additional Comments:

Fudge Points:

You can manually adjust the score by adding positive or negative points to this box.

Update Scores

**Final Score:** 37 out of 37

*loss of taste and smell can be removed to make the case less COVID-19 specific with a broader differential diagnosis.
