# Supplementary material for: Social Determinants of Health: A Multilingual Standardized Patient Case to Practice Interpreter Use in a Telehealth Visit
Source: MedEdPORTAL. 2023 Nov 14;19:11364. doi: 10.15766/mep_2374-8265.11364 (PMC10643468; doi:10.15766/mep_2374-8265.11364)
Supplement: Supplementary file 1 — SP Case - Spanish.docxSP Case - Tagalog.docxSP Case - Igbo.docxSP Case - French.docxSMI - Spanish.docxSMI - Tagalog.docxSMI - Igbo.docxSMI - French.docxSPL Rehearsal Script.docxDoor Instructions - Spanish and Tagalog.docxDoor Instructions - Igbo.docxDoor Instructions - French.docxFaculty Guide.pdfStudent Guide.pdfImportant Points Interpreters Telehealth.docxGraphic Instructional Tool.pdfSample Progress Note.docxProgress Note Grading Rubric.xlsx [file mep_2374-8265.11364-s001.zip › _Educational Summary Report_11364.pdf]

MedEdPORTAL® The AAMC Journal of Teaching and Learning Resources

Original Publication Open Access

Social Determinants of Health: A Multilingual Standardized Patient Case to Practice Interpreter Use in a Telehealth Visit

Gigi Guizado de Nathan, Laura K. Shaw, MD, Jessica Doolen, PhD, APRN, CNE, CHSE

Corresponding author: gigiguizado@gmail.com

Abstract

Introduction: The growing diversity of the United States population and strong evidence of disparities in health care make it critically important to educate health care professionals to effectively address issues of culture. To that end, we developed a simulation for teaching interpreter use in a telehealth setting. Our contribution of non-English language preference (NELP) patient cases in Spanish, Tagalog, French, and Igbo advances existing literature by combining the skills of interpreter use and telehealth while widening the array of cultures represented. **Methods:** Simulations were implemented for two cohorts of 60 first-year medical students. In the pilot, nine groups of six to seven students and one faculty met via Zoom with an NELP patient complaining of fatigue, weakness, and cough. When students determined the need for an interpreter, faculty admitted one to the meeting, and the telehealth visit continued. Postsession activities included debriefing and writing a progress note. **Results:** Course evaluation comments from the first cohort and a postencounter survey of the second cohort were positive. They revealed that students learned to speak slower, in shorter phrases, and directly to the patient. Learners completed note documentation according to a rubric. **Discussion:** This low-stakes activity provides faculty with a resource for introducing cultural competence into the curriculum. The original Spanish version of the case has been translated into three additional languages, providing a diverse representation of the NELP population. Important points for communicating through an interpreter are practiced in a telehealth setting with a fatigue case.

Keywords
Interpreter Communication, Non-English Language Preference, NELP, Telemedicine Competencies, Cultural Competence, Health Equity, Online/Distance Learning, Standardized Patient, Diversity, Equity, Inclusion, Telehealth, Language-Appropriate Health Care

Educational Objectives

By the end of this activity, learners will be able to:

1. Develop ways to create an environment conducive to conducting a telehealth visit that includes an interpreter.
2. Demonstrate appropriate history gathering and physical exam components while interviewing a patient with fatigue during a telehealth visit.
3. Apply techniques from the interpreter services reference materials to interview a non-English language preference patient with an interpreter and critique a peer after observing.
4. Integrate information from the case and faculty and peer feedback to create a progress note with an appropriate basic differential diagnosis and treatment plan for a patient with fatigue.

Citation:
Guizado de Nathan G, Shaw LK, Doolen J. Social determinants of health: a multilingual standardized patient case to practice interpreter use in a telehealth visit. *MedEdPORTAL*. 2023;19:11364.
https://doi.org/10.15766/mep_2374-8265.11364

Introduction

People of color and populations who speak English as a second language suffer a disproportionate burden of disease.[1] Southern Nevada is among the fastest growing and most diverse areas in the United States with significant unmet health needs. Its demographic profile in 2020 reflects what the US demographic is projected to look like in 2044.[2] Just over half the state is White (without also identifying as Latino), 29% is Hispanic, and 10% is African American, while Asian Americans are the fastest growing population at 9%.[3] Given this diverse demographic, it is not surprising that in Nevada, 31% of people speak a language other than English in the home.[4] The number one non-English language spoken in the state is Spanish, followed by Tagalog.

Copyright © 2023 de Nathan et al. This is an open-access publication distributed under the terms of the Creative Commons Attribution license.

Non-English language preference (NELP) is not limited to Nevada. In United States homes, over 350 languages are spoken.[5] In 2018, one in 15 people residing in the United States preferred a non-English language. Predictions are that this number will increase to 67 million by 2050. Nationally, less than 6% of US physicians identify themselves as Spanish speaking.[6] This number is concerning when there are a large number of Spanish-speaking individuals who need care and will be dependent on interpreters. The outlook for individuals with less common primary languages is even more dismal.

Medical interpreters serve a critical role in health care as mediators between NELP patients and primary care providers. Title VI of the Civil Rights Act of 1964 mandated federally funded health institutions to provide interpreter services for NELP patients.[7] The mandate is important because patients with language barriers demonstrate a poor follow-up rate and poorer health outcomes. As the NELP population in the US increases, so does the need for medical interpreters and bilingual medical staff. In contrast, the literature reports that medical interpreters are underutilized.[8] Some interpreters translate over the phone, and this is especially relevant in a pandemic context.[9,10] It is likely that NELP patients died of COVID-19 in hospitals without the ability to express their needs adequately due to language barriers.

The opportunity to address the aspect of provider proficiency with an interpreter in a telehealth setting arose when the University of Nevada, Las Vegas, School of Medicine suddenly transitioned to remote education. The curricular context for a lesson in working with interpreters was established prior to the pandemic with a previous cohort with other cases. Our work builds upon existing literature addressing health equity problems by expanding the use of interpreters in a telehealth setting to multiple languages, including Spanish, Tagalog, Igbo, and French.[11,12] These languages reflect the diverse populations in our area and those that disproportionately suffer negative outcomes.[4]

At the time of our project, little was known about how lack of interpreter use might impact COVID-19 outcomes for NELP patients. However, the disproportionate rate of exposure, infection, and death among people of color, identifying employment in essential jobs as a contributing factor, was suspected and has since been quantified.[13] This emerging information was woven into the case. In keeping with Association of Standardized Patient Educators (ASPE) Standards of Best Practice (SOBP) domain 2,[14] relevant subject matter experts were engaged in the creation of materials. By engaging translators fluent in each of the languages represented and adapting social history case details as appropriate, we accomplished ASPE SOBP 2.1.3, ensuring that cases were based on authentic problems and respecting the individuals represented in a case to avoid bias or stereotyping marginalized populations.[14]

Methods

This simulation was built upon first-year medical students' knowledge of the pulmonary interview and exam. It introduced students to the basics of working with interpreters. The simulation is appropriate for any first-year medical student, with only the prereadings required. Faculty and standardized patient (SP) educators did not have access to trained medical interpreters for use in this formative session. The provided materials enabled SP educators to utilize bilingual talent from their SP pool. By providing bilingual translated case materials (Appendices A-H), we enabled monolingual English-speaking SP trainers to work effectively with a multilingual cast. This fulfilled ASPE SOBP 3.1.2: Address one's own linguistic knowledge gaps, if any.[14] The translated case materials also helped mitigate the differences in dialect between speakers of a common language, satisfying ASPE SOBP 3.2.4: Ensure consistency and accuracy of role portrayal of individual SPs and among groups of SPs portraying the same role.[14]

The SP training materials were designed for two participants: the SP (Appendices A-D) and the simulated medical interpreter (SMI; Appendices E-H). The SP training materials contained all of the ASPE SOBP 2.2 case components in English, with spoken lines translated into Spanish, Tagalog, French, and Igbo (Appendix A-D).[14] The SMI training materials additionally included anticipated interview questions in English and in translation (Appendix E-H). This helped prepare the SMI for medical jargon and other phrases that may have been outside their layperson vocabulary.

For the pilot, we recruited eight bilingual Spanish/English speakers and two bilingual Tagalog/English speakers. The most fluent were assigned to the interpreter (SMI) role. The SPs and SMIs had two 3-hour rehearsals, fulfilling ASPE SOBP 3.2.5: Ensure SP readiness for the simulation activity through repeated practice.[14] The first rehearsal focused on case materials, the logistics of the session, and understanding the educational objectives. Five English-speaking simulated participants portraying learners (SPLs) were included in the second rehearsal. Scripted interview questions in English were provided (Appendix I), enabling the SPLs to simulate a realistic interview for the SMIs to practice interpreting.

Copyright © 2023 de Nathan et al. This is an open-access publication distributed under the terms of the Creative Commons Attribution license.

Faculty were given door instructions (Appendix J-L) and a faculty guide (Appendix M) with instructions in advance via email. Each group consisting of one SP, one SMI, one faculty, and six to seven students received a Zoom meeting link 1 week in advance. Expectations for the session were given via objectives in the student guide (Appendix N) posted in the university's learning management system, Canvas. Four presession readings were included: (1) a textbook chapter on fatigue,[15] (2) a tips sheet called "Important Points About Interpreters and Telehealth" (Appendix O), (3) the article "The Telehealth Ten: A Guide for Patient-Assisted Virtual Physical Examination,"[16] and (4) an optional graphic instructional tool (Appendix P).

All sessions of the pilot took place on the same morning. Faculty had Zoom accounts and were experienced in using them. Students and faculty participated from their homes. The SPs, SMIs, SP trainer, SP coordinator, and a sim lab technician worked from the SP suite in our clinical simulation center. Using laptop computers in individual rooms, SPs and SMIs positioned themselves in front of a blank wall. This achieved the look of an at-home telehealth appointment setting while providing the safety net of onsite SP program staff if technical support was needed. It also upheld ASPE SOBP domain 1: safe work environment.[14]

To start the session, the prebrief included a 10-minute review on the use of interpreters, an initial approach to a patient with fatigue, and which physical exam elements could be done virtually. The textbook chapter on fatigue[15] highlighted the pertinent review of systems and differential guidelines. Faculty selected a student or combination of students to be the interviewer in the simulation. Then, the faculty shared their screen, revealing the door instructions to all of the students. Next, the faculty admitted the patient (SP) from the Zoom waiting room, and the interview began. Once the interviewer noticed that they needed an interpreter, they could request one. The faculty then admitted the interpreter (SMI) from the waiting room, and the interview continued. The remaining students were encouraged to comment via the chat function when they observed elements of either proper or improper interpreter use, as well as elements related to fatigue or the telehealth environment, based on the reading materials.

A total of nine clinical faculty facilitated nine group sessions, consisting of six to seven students each, in the pilot, lasting 90 minutes. Each group completed the encounter once, with two students performing the interview. To serve all nine groups, SPs and SMIs repeated the session twice. We allowed 30 minutes for the encounter with the SP and SMI, 30 minutes for postencounter discussion, and 30 minutes for technical problems. For cohort 2, 10 faculty with five to six students per group repeated the case up to three times as we could better anticipate the timing and technical requirements (refer to the sample schedule in Appendix M).

After the encounter, learners were required to write a progress note (Appendix Q) in Canvas in SOAP (subjective, objective, assessment, plan) format. The recommendation was to complete this note in 10 minutes. A grading rubric (Appendix R) was created based on progress note items discussed in one of the course textbooks, *The Patient History: An Evidence Based Approach to Differential Diagnosis.*[17] Faculty graded the notes in Canvas via this Excel sheet rubric of 33 checklist items valued at 1 point each. The faculty used this Microsoft Excel rubric format repeatedly during the course for note grading due to the ease of point tallying and standardization. Faculty had attended meetings during the creation of the course to practice with the rubrics in order to avoid subjectivity as much as possible. Students received both written faculty comments and a rubric score. Seventy percent of the points or more were considered passing.

Because this module began as a quick pandemic pivot, no specific feedback comments had been planned for the pilot. Instead, feedback from our first cohort was excerpted from the Doctoring course evaluations. For the second cohort, we developed an anonymous feedback survey that was distributed at the time of the sessions with a link in Canvas. The survey included a total of 11 questions: eight questions scored on a 5-point Likert scale and three free-text response questions.

Results

Data from the progress note grading rubrics showed that cohort 1 (57 students) had a mean score of 84% correct on the rubric, with a range of 64% to 100%. Cohort 2 (52 students) showed even higher scores, with a mean of 94% and a range of 84% to 100%. Seventy percent was considered a passing score. Only three students in cohort 1 did not pass, whereas all students in cohort 2 passed.

Cohort 1 (57 Students)—Pilot

Course evaluations from students and verbal feedback from faculty showed an overwhelmingly positive response, with some suggestions for improvement. The interpreter SP encounters were highly valued by faculty and medical students. Students appreciated that "being able to interact with SPs over Zoom was more helpful than the asynchronous videos because it allowed

Copyright © 2023 de Nathan et al. This is an open-access publication distributed under the terms of the Creative Commons Attribution license.

students to experience interviewing patients." The interpreter case "helped medical students develop clinical skills that deal with social issues." The telehealth setting helped them realize that "many appointments can be done from home."

Cohort 2 (52 Students)

The participant postsurvey data (Table) clearly show there was a perception of a lack of competence and comfort when working with an interpreter prior to the session (questions 1 and 3). Students, SPs, and faculty strongly felt that interpreter skills were important to the practice of medicine (question 5). Results demonstrated that SP practice was considered beneficial for improving this perception of lack of competence and comfort and should be included in the medical school curriculum (questions 6 and 7). The data show that participants felt this session was effective in improving the comfort level of all participants and was presented in a clear and realistic way (questions 2, 4, and 8).

Themes emerged when examining the comments. The responses about lessons learned (question 9, $N = 48$ out of 64 responding) fell into three themes: the need to speak in shorter sentences and slow the pace, the need to speak directly to the patient, and the effect of interpreters on rapport. Themes also emerged on what students liked about the session: They liked the experience of practicing with an interpreter, how realistic it felt, the exposure to other languages, and the clinical content regarding COVID-19, and they expressed appreciation for the SPs. When students were asked if there were any objectives or case-related issues that could be improved (question 11, $N = 46$ out of 64 responding), the primary themes were that most felt no improvement was needed, while a few requested further guidance in general. Themes and representative quotes are listed below.

- Things learned:
  - Speaking in short phrases, slower pace: "I learned how to speak in short and concise sentences so that the interpreter is able to effectively communicate all of what I say to the patient."
  - Speaking directly to the patient: "Talk to the patient and not the interpreter. It seems common sense but actually doing it in practice is tough."
  - Influence of interpreter on rapport: "I learned that it is more difficult to show empathy for a patient when using a translator. It was not hard to care or show concern for what the patient was experiencing, but using the interpreter created a little barrier in translating the feelings through words. The emotional bounce was not as effective in the patient-physician interaction."
- Things liked:
  - Practicing with an interpreter: "I liked being put in the situation of talking to an interpreter. I didn't think it was going to be difficult, but it definitely was a learning curve. I'm glad I got this practice before having a situation like this happen in real life."
  - Realistic: "It seemed realistic and was a part of medicine that I never really thought about before today!"
  - Exposure to languages: "I liked that the patient spoke a completely foreign language that I could not understand."

Table. Postsession Survey of All Participants ($N = 64$)

Question	Mean Score				
	Students ($N = 51$)	SPs ($N = 3$)	SPIs ($N = 4$)	SPEs ($N = 2$)	Faculty ($N = 4$)
1. Self-rating of competence in using an interpreter in the context of telehealth.[a]	1.4	2.0	2.3	4.0	3.3
2. Agreement with the statement: The objectives of this session were clear.[b]	4.2	4.7	4.0	4.0	4.8
3. Comfort level PRIOR to the session for participating in situations where an interpreter is required in a telehealth setting.[c,d]	2.9	3.3	4.0	4.5	4.3
4. Comfort level AFTER the session for participating in situations where an interpreter is required in a telehealth setting.[c]	4.2	4.7	4.8	5.0	4.8
5. Agreement with the statement: The interpreter skills required in the case are important to the practice of medicine.[b]	4.9	5.0	5.0	5.0	4.5
6. Agreement with the statement: Simulated SP sessions with interpreters are helpful prior to actual patient encounters.[b]	4.7	5.0	5.0	5.0	4.5
7. Agreement with the statement: Simulated interpreter cases should be a mandatory part of the curriculum for medical students.[b]	4.7	5.0	5.0	5.0	4.0
8. Agreement with the statement: The case seemed realistic and representative of something one might see as a real patient encounter.[b]	4.6	4.3	5.0	5.0	4.5

Abbreviations: SP, standardized patient; SPE, SP educator; SPI, SP interpreter.
[a]Rated on a 4-point scale (1 = *No experience*, 2 = *Beginner*, 3 = *Approaching competence*, 4 = *Experienced*).
[b]Rated on a 5-point scale (1 = *Not at all*, 2 = *Not much*, 3 = *Neutral*, 4 = *Somewhat*, 5 = *Very much*).
[c]Rated on a 5-point scale (1 = *Not comfortable at all*, 2 = *Not very comfortable*, 3 = *Neutral*, 4 = *Somewhat comfortable*, 5 = *Very comfortable*).
[d]$N = 62$; two responses were left blank.

Copyright © 2023 de Nathan et al. This is an open-access publication distributed under the terms of the Creative Commons Attribution license.

    - SP appreciation: "Our interpreter was amazing and made the entire patient encounter very comforting, especially since we were nervous about having to deal with an interpreter!"
- Areas for improvement:
    - Objectives/case related: "None that I felt needed improvement."
    - Further guidance: "Maybe have a recording of an excellent example to guide our questioning from. It could help to have some frame of reference on how fast the conversation needs to be."

Based on faculty and staff debriefing of the pilot and feedback from cohort 1, some refinements were made for use with cohort 2: The faculty guide (Appendix M) was improved with written guidelines for facilitating the encounter, the schedule was streamlined to allow for more student participation, and SPs and SMIs were included in the postencounter discussions with cohort 2.

Discussion

The value of this educational activity is its ability to foreground a population that has been socially marginalized and to practice the skills urgently needed to close a gap in care. The unique contributions of SP and SPI case materials in Spanish, Tagalog, French, and Igbo were carefully crafted to represent a broad range of NELP patients.

From an SP educator perspective, the challenge with this simulation is finding people with the language skills to portray the patient and interpreter roles. It is helpful to include a question about languages spoken in the recruitment process for one's program in general. Ask coworkers and other colleagues if they speak another language or have friends or family members who do. Network with other SP programs. This is a telehealth scenario, which opens the possibility of working with SPs outside of one's immediate area. If an SP is unsure whether their bilingual proficiency is adequate to be successful in this activity, sharing the script with them during the recruitment process can be a helpful tool to clarify skill level. Recruiting bilingual SPs and getting to know them can be highly rewarding. It leads to a deeper understanding of people, cultures, and languages, while shedding light on social determinants of health and underserved populations that may be going unnoticed in the curriculum. For this reason, people with bilingual skills are motivated to participate as SPs and SPIs. They enjoy being part of the solution.

From a faculty perspective, this activity helps achieve the AAMC cultural competence requirements.[18] It provides faculty with an organized means of introducing cultural competence into their curriculum, whereby the important points for communicating through an interpreter are encountered in a telehealth setting. Our faculty appreciated the innovation of going beyond Spanish interpreters, recognizing that there were other languages needing translation. They found the simulation realistic and helpful and liked how it gave students an important opportunity to practice. Feedback from the first cohort, as well as the need for extra time when using an interpreter, led to refinement of the schedule, allowing more learners to participate during the second implementation of the case a year later (Appendix M).

Upon reflecting on the limitations of the case, we had concerns that we did not have access to professional medical interpreters for this simulation. We explored this limitation by including the SMIs in the survey. They confirmed it would be beneficial to include professional medical interpreters if at all possible. Also, due to the initial learning curve of transitioning from in-person to telehealth simulation, the complexity of content in multiple languages, and the inclusion of interpreters, we chose not to include an SP checklist. Development of checklists for SPs and SMIs in this simulation is an area for future improvement and would be a valuable addition.

One insight gained from developing and piloting this activity is that additional effort may be required to include African diaspora representation. Therefore, the description of race in the case demographics is intentionally broad, and an Igbo translation has been included, as Nigeria is the largest source of African immigration to the United States of America.[19]

Since the successful pilot, we have implemented this activity with a smaller staff of one SP trainer and SPs who work from home. Future opportunities for the activity include translating it into additional languages. Translation into any language is possible with just two people who are fluent in a common language to translate the materials and play the SP and SMI roles.

Appendices

A. SP Case - Spanish.docx
B. SP Case - Tagalog.docx
C. SP Case - Igbo.docx
D. SP Case - French.docx
E. SMI - Spanish.docx
F. SMI - Tagalog.docx
G. SMI - Igbo.docx
H. SMI - French.docx

Copyright © 2023 de Nathan et al. This is an open-access publication distributed under the terms of the Creative Commons Attribution license.

I. SPL Rehearsal Script.docx

J. Door Instructions - Spanish and Tagalog.docx

K. Door Instructions - Igbo.docx

L. Door Instructions - French.docx

M. Faculty Guide.pdf

N. Student Guide.pdf

O. Important Points Interpreters Telehealth.docx

P. Graphic Instructional Tool.pdf

Q. Sample Progress Note.docx

R. Progress Note Grading Rubric.xlsx

All appendices are peer reviewed as integral parts of the Original Publication.

Gigi Guizado de Nathan: Former Standardized Patient Coordinator, Clinical Simulation Center of Las Vegas, University of Nevada, Las Vegas; ORCID: https://orcid.org/0000-0002-1261-5577

Laura K. Shaw, MD: Associate Professor, Kirk Kerkorian School of Medicine at University of Nevada, Las Vegas

Jessica Doolen, PhD, APRN, CNE, CHSE: Associate Professor in Residence, School of Nursing, University of Nevada, Las Vegas; ORCID: https://orcid.org/0000-0002-5482-7682

Acknowledgments
We thank translators Brenda Lopez, Marie Christine Padilla, BSHCA, Ogonnaya Onyema, DNP, RN, CMSRN, and Steven Fehr, MFA; standardized patient educators Brandi Blackman and Timothy Cummings; and Aditi Singh, MD.

Disclosures
None to report.

Funding/Support
None to report.

Prior Presentations
Guizado de Nathan G, Doolen J. Social determinants of health. Presented virtually at: Annual Meeting of the Association of Standardized Patient Educators (ASPE); June 27-30, 2021.

Ethical Approval
Reported as not applicable.

References

1. Hill L, Ndugga N, Artiga S. Key data on health and healthcare by race and ethnicity. KFF. March 15, 2023. Updated March 29, 2023. Accessed October 10, 2023. https://www.kff.org/racial-equity-and-health-policy/report/key-data-on-health-and-health-care-by-race-and-ethnicity/
2. Kolko J. 40 years from now, the U.S. could look like Las Vegas. FiveThirtyEight. June 22, 2017. Accessed October 10, 2023. https://fivethirtyeight.com/features/40-years-from-now-the-u-s-could-look-like-las-vegas/
3. Quick Facts: Clark County, Nevada. United States Census Bureau. Accessed October 10, 2023. https://www.census.gov/quickfacts/clarkcountynevada
4. State immigration data profiles: United States: Nevada: language & education. Migration Policy Institute. Accessed October 10, 2023. https://www.migrationpolicy.org/data/state-profiles/state/language/US/NV/
5. Census Bureau reports at least 350 languages spoken in U.S. homes. United States Census Bureau. November 3, 2015. Updated December 16, 2021. Accessed October 10, 2023. https://www.census.gov/newsroom/archives/2015-pr/cb15-185.html
6. Abuelo C. The U.S. needs more Spanish-speaking doctors. U.S. News. August 25, 2020. Accessed October 10, 2023. https://www.usnews.com/news/healthiest-communities/articles/2020-08-25/why-we-need-more-spanish-speaking-doctors
7. Civil Rights Act of 1964, Pub L No. 88-352, 78 Stat 241.
8. Neira L. The importance of addressing language barriers in the US health system. Duke Center for Personalized Health Care. July 17, 2018. Accessed October 10, 2023. https://personalizedhealth.duke.edu/blog/importance-addressing-language-barriers-us-health-system
9. Karliner LS, Jacobs EA, Chen AH, Mutha S. Do professional interpreters improve clinical care for patients with limited English proficiency? A systematic review of the literature. *Health Serv Res.* 2007;42(2):727-754. https://doi.org/10.1111/j.1475-6773.2006.00629.x
10. Murphy JE, Washington D, Xuan Z, Paasche-Orlow MK, Drainoni ML. Identifying and addressing language needs in primary care: a pilot implementation study. *J Racial Ethn Health Disparities.* 2019;6(3):505-516. https://doi.org/10.1007/s40615-018-00549-6
11. Pinto Taylor E, Mulenos A, Chatterjee A, Talwalkar JS. Partnering with interpreter services: standardized patient cases to improve communication with limited English proficiency patients. *MedEdPORTAL*. 2019;15:10826. https://doi.org/10.15766/mep_2374-8265.10826
12. Afonso N, Kelekar A, Alangaden A. “I have a cough”: an interactive virtual respiratory case-based module. *MedEdPORTAL*. 2020;16:11058. https://doi.org/10.15766/mep_2374-8265.11058
13. National Center for Immunization and Respiratory Diseases; Advisory Committee on Immunization Practices. Risk for COVID-19 infection, hospitalization, and death by race/ethnicity.

Copyright © 2023 de Nathan et al. This is an open-access publication distributed under the terms of the Creative Commons Attribution license.

Centers for Disease Control and Prevention. Updated April 23, 2021. Accessed October 10, 2023. https://stacks.cdc.gov/view/cdc/105453

14. Lewis KL, Bohnert CA, Gammon WL, et al. The Association of Standardized Patient Educators (ASPE) Standards of Best Practice (SOBP). *Adv Simul (Lond)*. 2017;2:10. https://doi.org/10.1186/s41077-017-0043-4
15. Simmons RJ, Swallow NA. Fatigue. In: Henderson MC, Tierney LM Jr, Smetana G, eds. *The Patient History: An Evidence-Based Approach to Differential Diagnosis.* 2nd ed. McGraw-Hill; 2012:47-56.
16. Benziger CP, Huffman MD, Sweis RN, Stone NJ. The Telehealth Ten: a guide for patient-assisted virtual physical examination. *Am J Med.* 2021;134(1):48-51. https://doi.org/10.1016/j.amjmed.2020.06.015
17. Henderson MC, Tierney LM Jr, Smetana G, eds. *The Patient History: An Evidence-Based Approach to Differential Diagnosis.* 2nd ed. McGraw-Hill; 2012.
18. Colleges Association of American Medical. *Cultural Competence Education*. Association of American Medical Colleges; 2005. Accessed October 10, 2023. https://www.aamc.org/media/20856/download
19. *RAD Diaspora Profile: The Nigerian Diaspora in the United States.* Migration Policy Institute; 2015. Accessed October 10, 2023. https://www.migrationpolicy.org/sites/default/files/publications/RAD-Nigeria.pdf

Received: November 14, 2022
Accepted: September 14, 2023
Published: November 14, 2023

Copyright © 2023 de Nathan et al. This is an open-access publication distributed under the terms of the Creative Commons Attribution license.
